# Ion Separations Based on Spontaneously Arising Streaming Potentials in Rotating Isoporous Membranes

**DOI:** 10.3390/membranes12060631

**Published:** 2022-06-18

**Authors:** Chao Tang, Andriy Yaroshchuk, Merlin L. Bruening

**Affiliations:** 1 Department of Chemical and Biomolecular Engineering, University of Notre Dame, Notre Dame, IN 46656, USA; ctang2@nd.edu; 2 ICREA, pg.L.Companys 23, 08010 Barcelona, Spain; andriy.yaroshchuk@upc.edu; 3 Polytechnic University of Catalonia, Av. Diagonal 647, 08028 Barcelona, Spain; 4 Department of Chemistry and Biochemistry, University of Notre Dame, Notre Dame, IN 46556, USA

**Keywords:** streaming potential, ion separations, selectivity

## Abstract

Highly selective ion separations are vital for producing pure salts, and membrane-based separations are promising alternatives to conventional ion-separation techniques. Our previous work demonstrated that simple pressure-driven flow through negatively charged isoporous membranes can separate Li^+^ and K^+^ with selectivities as high as 70 in dilute solutions. The separation mechanism relies on spontaneously arising streaming potentials that induce electromigration, which opposes advection and separates cations based on differences in their electrophoretic mobilities. Although the separation technique is simple, this work shows that high selectivities are possible only with careful consideration of experimental conditions including transmembrane pressure, solution ionic strength, the K^+^/Li^+^ ratio in the feed, and the extent of concentration polarization. Separations conducted with a rotating membrane show Li^+^/K^+^ selectivities as high as 150 with a 1000 rpm membrane rotation rate, but the selectivity decreases to 1.3 at 95 rpm. These results demonstrate the benefits and necessity of quantitative control of concentration polarization in highly selective separations. Increases in solution ionic strength or the K^+^/Li^+^ feed ratio can also decrease selectivities more than an order of magnitude.

## 1. Introduction

Highly selective ion separations are important for recovering pure salts from natural resources or secondary waste streams [1]. For example, geothermal brines contain a high concentration of Li^+^ (compared to seawater) and are a key source of battery-grade Li_2_CO_3_ (99.5 wt% purity) [2,3]. Nevertheless, these brines contain large excesses of Na^+^, K^+^, Ca^2+^, Mg^2+^, and other cationic species, therefore Li_2_CO_3_ production from brines is a challenging ion-separation process [4].

Conventional ion separations typically employ chemical precipitation, solvent extraction, ion exchange, or a combination of these techniques [5,6]. Although these established processes yield highly pure salts, they often have negative environmental impacts due to high water usage, employment of harsh chemicals, and improper waste management [6]. In contrast, membrane-based ion separations may drastically reduce the environmental footprint of the separation process, but more selective membranes are needed [4] to produce highly pure salts.

Many studies have demonstrated that membranes can effectively separate monovalent and multivalent ions [7,8,9,10,11,12,13]. For example, membranes with a dense, positively charged polyelectrolyte coating showed remarkable Li^+^/Mg^2+^ selectivities around 1000, and electrodialysis with such membranes can recover 60% of the Li^+^ from a source-phase solution while creating 99.9% pure Li^+^ in the receiving phase [7]. The dense structure of the polyelectrolyte film greatly impedes Mg^2+^ migration because of its large hydrodynamic radius, and the positive surface charge also excludes multivalent Mg^2+^ more than singly charged Li^+^ (Donnan exclusion) [14,15].

Membrane-based separations among ions that have the same charge are more challenging than monovalent/multivalent ion separations, largely because Donnan exclusion does not offer selectivity among ions that have the same charge. Thus, researchers typically employ membranes with precisely defined pore sizes or specific ion-binding sites to separate these ions. Such membranes include layered graphene oxide sheets [16,17,18], metal organic frameworks [19,20], and crown-ether-functionalized polymers [21,22,23].

**Figure 1:**
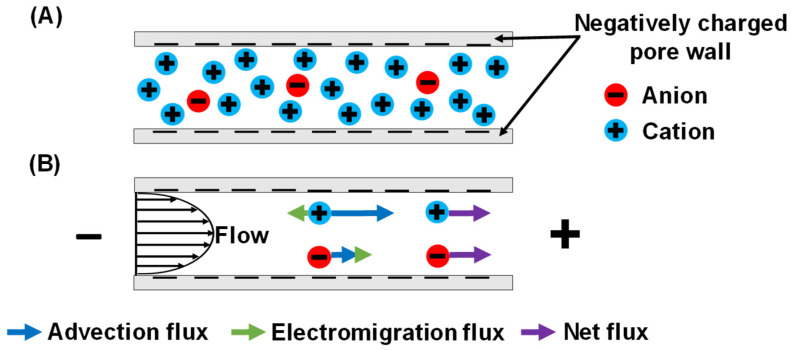
(**A**) Qualitative ion distribution in a negatively charged nanopore and (**B**) scheme of streaming potential and ion flux components during flow through the pore. Due to anion exclusion, the cation advection flux is much larger than the anion advection flux. The spontaneously arising streaming potential creates electromigration flux components, so that the net fluxes of cations and anions are equal (assuming that the cations and anions are both monovalent species) [24].

In addition to the above methods, under certain conditions simple pressure-driven flow in charged, isoporous membranes can separate ions that have the same charge but different electrophoretic mobilities. Our recent study based on this separation mechanism demonstrated Li^+^/K^+^ selectivities as high as 70 when flowing a dilute solution through polycarbonate track-etched membranes with 30-nm pores [24]. Remarkably, both selectivity and Li^+^ permeability can increase with transmembrane pressure, breaking the selectivity-permeability tradeoff. The separation mechanism relies on opposing advection with electromigration, which spontaneously arises in charged porous membranes provided that the solution ionic strength is sufficiently low. As Figure 1A shows, a negatively charged pore attracts cations and rejects anions, and pressure-driven flow through the pore carries more cations than anions via advection. In the absence of electrical current, the molar fluxes of cations and anions across the membrane must be equal (assuming they are both monovalent species). Thus, an electrical-potential difference spontaneously arises to induce electromigration to give zero current (Figure 1B) [25,26]. Note that flux is the product of concentration and velocity, so the net velocity of the depleted anion must be larger than that of the more abundant cation to create zero current.

**Figure 2:**
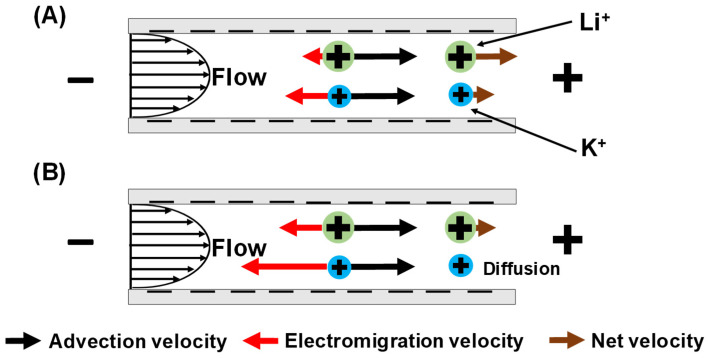
Scheme of Li^+^/K^+^ separation during flow through a negatively charged nanopore: (**A**) electromigration (red arrows) retards K^+^ more than Li^+^ due to the higher mobility of K^+^, whereas the advection velocities of both ions (black arrows) are the same; (**B**) with significantly strong streaming potentials, the magnitude of the K^+^ electromigration velocity exceeds its advection velocity, and K^+^ transports very slowly through the nanopore due to diffusion [24].

When Li^+^ and K^+^ are both present in the feed solution, the streaming potential induces different electromigration velocities for these two ions (Figure 2A). K^+^ has a larger electromigration velocity than Li^+^ because K^+^ has a smaller hydrodynamic radius, or equivalently a higher electrophoretic mobility [27]. Thus, Li^+^ has a larger net velocity through the membrane than K^+^, and a transmembrane flow leads to Li^+^/K^+^ separations. For sufficiently dilute feed solutions, the streaming potential is large enough to give a K^+^ electromigration velocity that completely overcomes its advection velocity (Figure 2B). This leads to very high Li^+^/K^+^ selectivities as the K^+^ flux approaches 0 at large flow velocities within the nanopores (see Section 3.3 for further discussion).

The earliest literature that proposes the above separation mechanism appeared 60 years ago [28,29,30], yet experimental results demonstrating high selectivities were not available until recently. As noted above, high Li^+^/K^+^ selectivities occur **when (1) the K^+^ electromigration velocity magnitude exceeds its advection velocity and (2) the flow velocity within the nanopore is sufficiently large such that diffusion does not greatly reduce selectivity**. These two necessary conditions impose a number of limitations on the choice of membrane and experimental conditions. For membranes that have pore diameters <10 nm, it is difficult to achieve sufficiently large flow velocities within the nanopores at transmembrane pressures below 60 bar (the high end of pressures employed in reverse osmosis). Condition (1) for high selectivity requires a high streaming potential drop across the nanopores. This limits the solution ionic strength to below 1 mM (Debye thickness ~10 nm) so that strong anion exclusion occurs in the pores. Aiming to satisfy both conditions (1) and (2), this work employs low ionic-strength solutions (below 1 mM) and hydrophilic polycarbonate track-etched membranes with 30 nm pores. The pore size distribution is narrow, and the pore surfaces are negatively charged due to the chemical-etching in the manufacturing process [26]. However, a challenge remains in controlling concentration polarization (CP, the accumulation of rejected solutes near the membrane surface). With severe CP, the solution ionic strength at the feed-solution/membrane interface can significantly increase to decrease streaming potentials. Selectivity condition (1) may no longer be satisfied at high CP due to weaker anion exclusion that decreases the streaming potential. The resulting relatively high K^+^ passage will decrease selectivity. The polycarbonate track-etched membranes employed in this work have around 1% porosity, but CP is still evident, particularly at low rotation rates and high transmembrane pressures (see Section 3.3).

To investigate the effects of CP on Li^+^/K^+^ separations, this work employs a recently developed rotating-membrane filtration cell to provide quantitative control of CP [31,32]. Compared to filtration cells that employ cross-flow or over-head stirring, the rotating cell has two advantages. First, the thickness of the CP layer varies with the membrane rotation rate, and one can estimate the boundary layer thickness. Additionally, the CP layer thickness is uniform over the membrane surface [33]. **Thus, for the first time, this study investigates the effects of CP on streaming potential-based separations and minimizes the effects of a non-uniform CP layer**. This work also examines the effects of feed-solution ionic strength and the Li^+^/K^+^ molar ratio in the feed on selectivities.

## 2. Materials and Methods

### 2.1. Materials

KCl (ACS reagent, 99.0–100.5%) was purchased from Sigma Aldrich (St. Louis, MO, USA), LiCl (crystal, ACS reagent) was obtained from Spectrum Chemical (New Brunswick, NJ, USA). K^+^ and Li^+^ standard solutions (TraceCert^®^, 1000 mg/L) were acquired from MilliporeSigma (Burlington, MA, USA). All solutions were prepared with deionized water (18.2 MΩ cm, Milli-Q Reference System from Millipore, Burlington, MA, USA,). Hydrophilic polycarbonate track-etched membranes (nominal 30 nm diameter pores, 47 mm disk diameter, 6 µm thickness) and nylon membranes (1.2 µm pore size, 47 mm disk diameter) were obtained from Sterlitech (Auburn, WA, USA). The track-etched membranes have a pore density of 6 × 10^12^ pores/m^2^ with a tolerance of ± 15%, according to manufacturer, and should have a nominal porosity around 0.4%. However, porosity calculations based on water permeabilities from a previous study [24] and from this work suggest that either the actual porosity is around 1% or that pores may have a “toothpick”-shaped structure with an effective diameter larger than 30 nm [34]. The burst strength of the track-etched membranes is 0.7 bar, per the manufacturer.

### 2.2. Rotating-Membrane Filtration Cell

**Figure 3:**
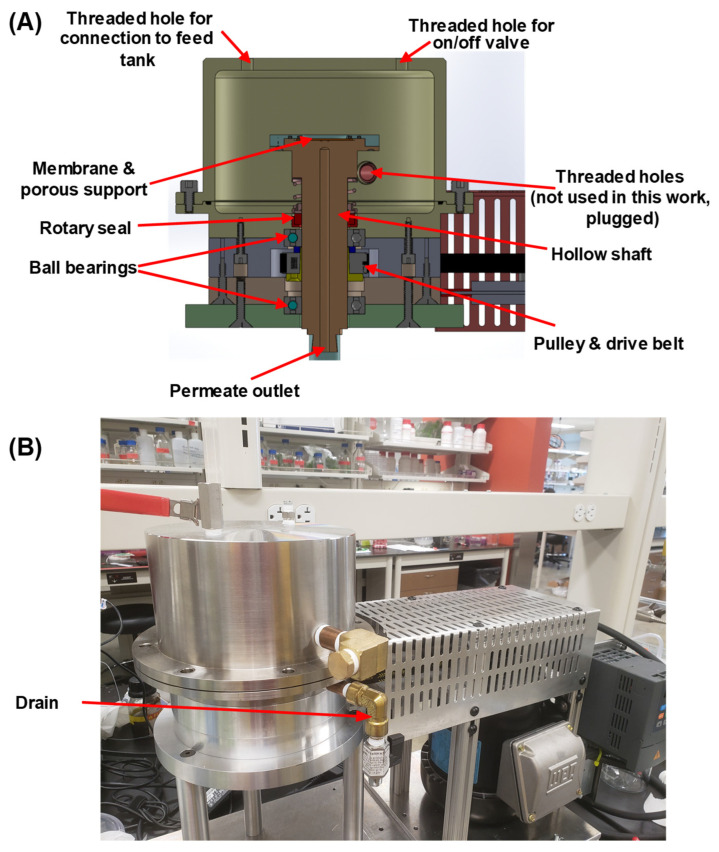
(**A**) Schematic diagram of the rotating-membrane filtration unit. The drain is not drawn in (**A**) but is shown in the photograph of the test unit (**B**).

The rotating-membrane filtration cell design is similar to those in previous works [31,35], and cell manufacturing was undertaken in the machine shop of the University of Notre Dame Physics department. Figure 3A shows the schematic diagram. The unit consists of a motor, drive belt, and cell body including the hollow rotating shaft. The motor has a variable frequency drive, and the shaft rotating speed can be adjusted from 95 to 2000 rpm, measured by a digital tachometer. The cell can hold 3 L of feed solution, and the exposed membrane area has a diameter of 4 cm. However, the membrane area near the o-ring seal should have less effective mixing and more severe concentration polarization [24,31] compared with membrane areas away from the o-ring seal. Thus, we taped the membrane with a donut-shaped “mask” to cover the membrane area near the o-ring. The effective membrane area after taping was 6.6 cm^2^ (2.9 cm diameter). The polycarbonate track-etched membrane rests on top of a nylon membrane, and this “composite” system sits on top of a porous stainless-steel frit that serves as a mechanical support. The nylon membrane prevents direct contact between the thin track-etched membrane and rough stainless-steel surface and minimizes membrane deformation under high pressures. The housing can withstand pressure as high as 10 bar, and a rotary seal prevents solution leakage while allowing the hollow shaft to rotate as fast as 2000 rpm. During operation, the rotary seal generates heat due to friction with the rotating shaft and can increase the fluid temperature from 21 to 29 °C in around 90 min at a 1000 rpm rotation rate. However, most experiments finished in under an hour, and the small increases in temperature during experiments were insignificant.

### 2.3. Filtration Setup

**Figure 4:**
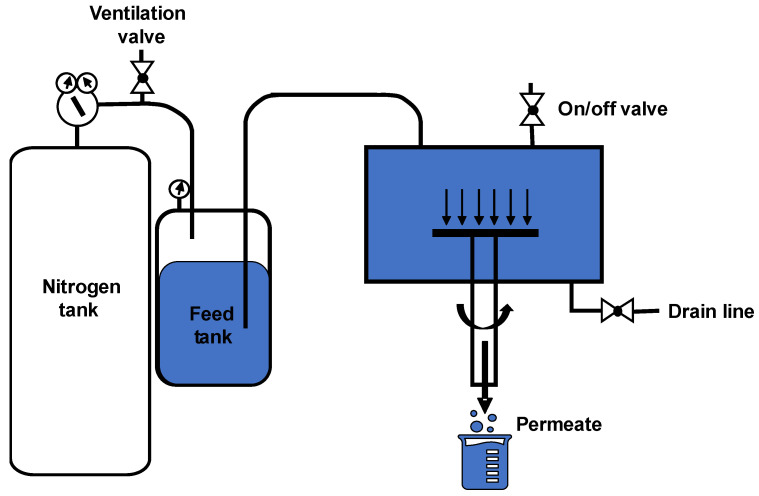
Schematic drawing of the filtration setup. Figure S10 in the supplementary materials provides a photograph of the experimental apparatus.

Figure 4 shows the scheme of the filtration apparatus. The fully assembled rotating-membrane cell is connected to a feed tank (3-gallon portable pressure vessel from Pope Scientific). The feed tank is connected to a nitrogen tank, which has a pressure-regulator valve and a ventilation valve. Prior to any experiments, the drain line of the filtration cell is closed, and the on/off valve is open. The feed tank is filled with 6 L of feed solution, and the pressurized nitrogen slowly pushes the feed solution from the feed tank to the filtration cell. Once the filtration cell is fully filled, feed solution overflows through the on/off valve, and the on/off valve is then immediately shut off. The filtration setup is ready for experiment.

During experiments, the system pressure or transmembrane pressure is controlled by the pressure-regulator valve on the nitrogen supply, and the transmembrane pressure gives permeate flow. When experiments are finished, the nitrogen supply is shut off, and the system is vented to atmospheric pressure by opening the ventilation value on the nitrogen tank. Subsequently, both the on/off valve and the drain line are opened, and the filtration cell is emptied. A typical experiment collects less than 30 mL of permeate, and the filtration cell contains 3 L of feed solution. Assuming 100% salt rejection, the salt concentration in the feed solution increases less than 1% in the test cell during one experiment, so we neglect such changes in data analysis.

Compared to our previous work [24], the above filtration setup has two clear advantages. First, the rotating-membrane cell should theoretically yield a uniform concentration polarization (CP) layer. In contrast, our prior work employed a dead-end filtration cell (Sterlitech HP4750, Auburn, WA, USA) with overhead stirring that gives a non-uniform CP layer. Thus, this work can investigate the effects of CP more systematically by ignoring the contribution of non-uniformity, which is particularly important if the separation is sensitive to the CP layer thickness. Moreover, the filtration cell in this work houses a lot more feed solution (3 L) than a Sterlitech HP4750 cell (less than 300 mL), so changes in feed solution concentrations should have minimal effect.

### 2.4. Permeability Measurements

Membrane-permeability measurements give important insights into the extent of concentration polarization, membrane porosity, and electroosmosis. Moreover, these measurements provide the flow velocity values in boundary layers in numerical simulations (see Section 2.6). The permeability is measured by the “bucket and stopwatch” method: at a given transmembrane pressure, the time to collect 5 mL of permeate is recorded, and the superficial velocity is calculated by dividing the permeate volume (5 mL) by the effective membrane area (6.6 cm^2^) and collection time. The transmembrane pressure varied from 1.4 bar (20 psi) to 6.9 bar (100 psi), and the superficial velocity at a given pressure was measured three times with a pristine membrane each time. The feed solution was 0.1 mM KCl. Additionally, the permeability measurements were also performed with 10 mM KCl to gauge the extent of electroosmosis and electroviscosity. All superficial velocities were measured with a membrane rotation rate of 1000 rpm.

### 2.5. Single-Salt and Mixed-Salt Filtration

In single-salt filtration, the aqueous feed solutions contain either KCl or LiCl at various ionic strengths. The permeate ion concentrations were determined using inductively coupled plasma optical emission spectroscopy (ICP-OES, Avio 200, Perkin-Elmer, Waltham, MA, USA) with calibration curves. Salt passages were calculated using Equation (1), and results are the average values from three pristine membranes. Uncertainties represent standard deviations.

(1) $$Passage\;\%=\frac{Permeate\;concentration}{Feed\;concentration}\times 100\%$$

In mixed-salt filtration, the feed solutions contain a mixture of K and Li salts at various Li^+^/K^+^ ratios. The ion passages are calculated with Equation (1), and the Li^+^/K^+^ selectivities are calculated using Equation (2). Again, experimental results are the average values from three pristine membranes and uncertainties are standard deviations.

(2) $$Selectivity=\frac{Li\;passage\;\%}{K\;passage\;\%}$$

In all studies, the first 15 mL of permeate was discarded because it was likely contaminated with residual solution in the dead volume of the test unit. The next 5 mL of the permeate was collected as a sample.

### 2.6. Numerical Simulations

This section describes only the framework and governing equations of numerical equations, interested readers can find the full details in the supporting information of our previous work [24], which employs the same numerical procedures and frameworks to model ion transport. The simulations employ the extended Nernst–Planck equation, Equation (3), to describe ion transport through the nanopores inside the track-etched membranes.

(3) $$j_{i}\left(r\right)=-D_{i}\frac{\partial c_{i}\left(x,r\right)}{\partial x}-z_{i}c_{i}\left(x,r\right)D_{i}\frac{F}{RT}\frac{\partial \phi \left(x,r\right)}{\partial x}+c_{i}\left(x,r\right)j_{v}\left(r\right)$$

In the above equation, $j_{i}$ is the ion flux inside the nanopore. $D_{i}$ and $z_{i}$ are the diffusion coefficient and the charge, respectively, for ion i. $c_{i}$ is the ion concentration, which depends on the axial coordinate, x, and radial coordinate, r. Additionally, ϕ is the electrostatic potential, $j_{v}$ is the flow velocity (volume flux) inside the pores, and F, R, and T are Faraday’s constant, the gas constant, and temperature, respectively. The first term on the right-hand-side of the equation describes diffusive flux governed by Fick’s law; the second term is the electromigration flux employing the Einstein–Smoluchowski relation between diffusivity and electrophoretic mobility; and the third term describes advection flux.

The polycarbonate track-etched membrane pores bear negative surface charge. Therefore, the ion concentrations within the nanopores also depend on the radial coordinate, and the simulations employ the Poisson–Boltzmann equation to model the ion distribution.

(4) $$\frac{\partial^{2}\phi \left(x,r\right)}{\partial r^{2}}+\frac{1}{r}\frac{\partial \phi \left(x,r\right)}{\partial r}=-\frac{F}{\varepsilon_{o}\varepsilon_{p}}\underset{i}{\sum }c_{i}\left(x,r\right)z_{i}.\;$$

In the above equations $\varepsilon_{o}\varepsilon_{p}$ is the dielectric permittivity of solution in the membrane pore.

Additionally, the simulation also assumes zero-current (Equation (5)), electroneutrality at the axial coordinate (Equation (6)), and the Hagen–Poiseuille equation for flow velocity (Equation (7)).

(5) $$0=F\underset{i}{\sum }z_{i}\int_{0}^{R_{p}}\frac{2\pi }{\pi R_{p}{}^{2}}j_{i}\left(r\right)rdr$$

(6) $$\left|\frac{2\pi R_{p}L\sigma }{\pi R_{p}{}^{2}LF}\right|=\underset{i}{\sum }z_{i}\int_{0}^{R_{p}}\frac{2\pi }{\pi R_{p}{}^{2}}c_{i}\left(x,r\right)rdr.\;$$

(7) $$j_{v}\left(r\right)=\frac{\Delta PR_{p}{}^{2}}{4\mu L}\left(1-\frac{r^{2}}{R_{p}{}^{2}}\right)$$

In the above equations, $R_{p}$ is pore radius, L is the pore length, and σ is the surface charge density on the pore wall. ΔP is the transmembrane pressure, and μ is the fluid dynamic viscosity.

To model ion transport within the boundary layer next to the membrane surface, this work again employs the extended Nernst–Planck equation.

(8) $$j_{i}{}^{\prime }=-D_{i}\frac{dc_{i}{}^{\prime }}{dx}-z_{i}c_{i}{}^{\prime }D_{i}\frac{F}{RT}\frac{d\phi }{dx}+c_{i}{}^{\prime }j_{v}{}^{\prime }$$

In this equation, $j_{i}{}^{\prime }$ is the ion flux within the boundary layer with respect to the whole membrane area (not just the area that has open pores), and $j_{i}{}^{\prime }$ is about 100 times smaller than the “mixing cup” value of $j_{i}$ due to the low membrane porosity. Equation (9) describes $j_{i}{}^{\prime }$, and the membrane porosity ε is around 1%, as calculated from water-permeability measurements and Equations (7) and (10). Similarly, $j_{v}{}^{\prime }$ is the flow velocity calculated with respect to the whole membrane area. $c_{i}{}^{\prime }$ is the ion concentration within the boundary layer.

(9) $$j_{i}{}^{\prime }=\varepsilon \int_{0}^{R_{p}}\frac{2\pi }{\pi R_{p}{}^{2}}j_{i}\left(r\right)rdr$$

(10) $$j_{v}{}^{\prime }=\varepsilon \int_{0}^{R_{p}}\frac{2\pi }{\pi R_{p}{}^{2}}j_{v}\left(r\right)rdr=\varepsilon \frac{\Delta PR_{p}{}^{2}}{8\mu L}$$

At the membrane/feed and membrane/permeate interfaces, this work employs a Donnan partitioning model [14].

(11) $$\frac{\int_{0}^{R_{p}}\frac{2\pi }{\pi R_{p}{}^{2}}c_{i}\left(r\right)rdr}{c_{i}{}^{\prime }}=exp\left(-\frac{z_{i}F\phi_{D}}{RT}\right)$$

where $\phi_{D}$ is the Donnan potential at the membrane/feed or membrane/permeate interfaces. The value of $\phi_{D}$ varies with ion fluxes, flow velocity, and surface charge density.

Finally, the permeate concentrations are simply the ion fluxes divided by the flow velocity (volume flux).

(12) $$c_{i,\;permeate}=\frac{\int_{0}^{R_{p}}\frac{2\pi }{\pi R_{p}{}^{2}}j_{i}\left(r\right)rdr}{\frac{\Delta PR_{p}{}^{2}}{8\mu L}}=\frac{j_{i}{}^{\prime }}{j_{v}{}^{\prime }}$$

Table 1 shows the parameters employed in the simulations. Our MATLAB numerical procedures can calculate the ion passages and fluxes at various flow velocities (pressures) and boundary-layer thicknesses.

The simulations reported in the body of the paper assume pore diameters of 30 nm, which is the nominal diameter provided by the manufacturer. Additionally, these simulations employ Equation (13), the Levich equation [33], for the boundary layer thicknesses (see Section S1 of the supplementary materials for justifications).

(13) $$\delta =1.61D_{i}{}^{1/3}\omega^{-0.5}{\left(\frac{\mu }{\rho }\right)}^{1/6}$$

In the above equation, δ is the boundary layer thickness, ω is the membrane rotation rate in rad/s, and ρ is the fluid density of 1000 kg/m^3^ at 25 °C. The surface charge density is assumed to be −2.2 mC/m^2^, which is on the order of prior measured results [24] and gives good agreement between data and simulations in single-salt studies. (A previous study reported a surface charge of −3.4 mC/m^2^ in a 1 mM KCl solution, but this work employs more dilute solutions so the surface charge could be lower [15]). As Section 3 shows, simulation results conform largely with trends in experimental data, particularly in single-salt studies, and give insights into the transport phenomenon. However, significant deviations between experimental data and simulations occur at high flow velocities in mixed solutions. This is expected for several reasons.

**Figure 5:**
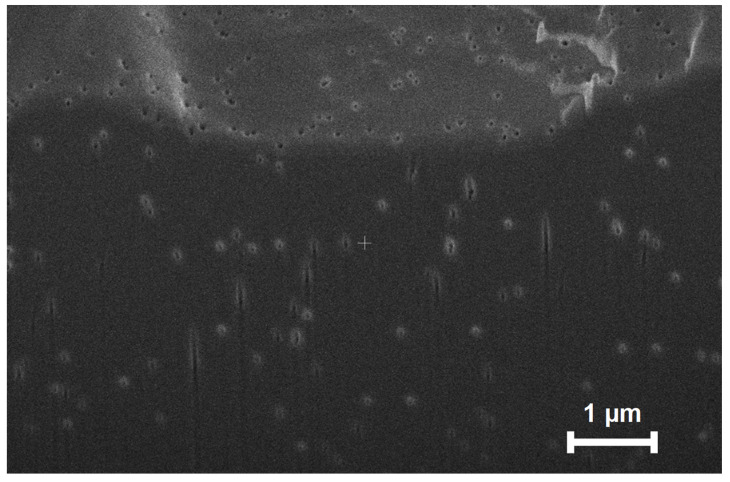
Cross-sectional SEM image of a polycarbonate track-etched membrane with 30 nm pores. The dark region is the cross section, whereas the brighter section at the top is the membrane surface. A focused ion beam was used to section the membrane sample in the middle prior to SEM imaging of the membrane cross section (Helios G4 Ux Dual Beam instrument). Prior to imaging, the membrane was coated with a layer of Palladium. Some pores show their tracks passing through the membrane, but most pores only show an entrance in the cross-section, and this indicates that pores have different orientations and different pore lengths.

First, the simulation assumes every pore path is perpendicular to the membrane surface and has the same length as the nominal membrane thickness, whereas SEM images of a membrane cross section show that pores paths are somewhat oblique or have different angles (Figure 5, Figures S1 and S9 the supplementary materials show more SEM images.) Additionally, the model does not consider possible changes in surface charge density at different ionic strengths [15]. Significant pore clustering could occur in some membrane areas and increase the flow velocity and extent of CP locally. Finally, if the tape is not a perfect mask, significant CP could occur near the edge of the tape. As an approximation, we also modelled the experimental data in this work using a boundary layer thickness that is 1.9 times what the Levich equation predicts. We present these modelling results in the supplementary materials (Figures S2–S6). At high pressures, the fits between experimental results and simulations agree better with the higher boundary layer thickness, but we do not have quantitative justification for the factor of 1.9.

## 3. Results and Discussion

This work aims to better understand the mechanism of ion separations that rely on spontaneously arising streaming potentials in negatively charged nanoporous membranes. Although the streaming potential phenomenon has been well studied [25,26,36,37,38], its effects on ion passages are complicated due to salt partitioning (more details below) that depends on CP. This study uses a rotating membrane to systematically vary CP and examine its effects on ion transport. In the following sections, we first employ permeability measurements to verify the presence of the streaming potential (and electroosmosis) during pressure-driven flow in negatively charged 30 nm nanopores. We then employ single-salt studies combined with numerical simulations to investigate the effects of streaming potentials on salt passages, with an emphasis on the interplay between ion partitioning and counter-flow electromigration. Section 3.3 examines the more complicated case in which the feed solution contains two salts. This section provides experimental and simulation results to examine the effect of CP on the ion-separation mechanism. We also discuss limitations of the separation mechanism.

### 3.1. Peameability Measurements and Electroosmosis

**Figure 6:**
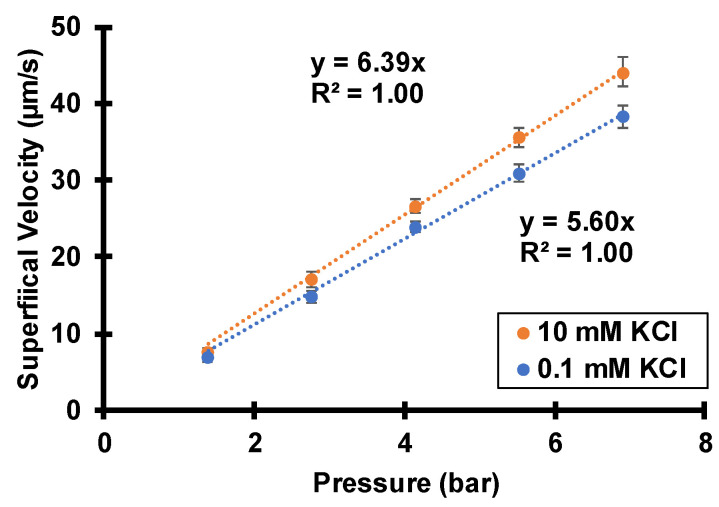
Superficial velocities of either 0.1 mM KCl or 10 mM KCl feed solutions passing through track-etched membranes (30 nm pores) at various transmembrane pressures. The membrane rotation rate is 1000 rpm.

Figure 6 shows the measured superficial solution velocities through track-etched membranes exposed to various transmembrane pressures. We define the superficial flow velocity as the volumetric flow rate divided by the entire exposed membrane area, 6.6 cm^2^. The actual solution velocity inside the 30-nm pores is two orders magnitude higher due to the 1% porosity of the membranes. With either 0.1 mM KCl or 10 mM KCl as test solutions, the superficial velocities increase linearly with transmembrane pressures, as expected from the Hagen–Poiseuille model.

Interestingly, the solution permeability with 10 mM KCl is about 15% higher than with 0.1 mM KCl. At first, this seems counter-intuitive: the Hagen–Poiseuille equation suggests that the permeability should be slightly smaller with the more concentrated solution because of a modest increase in viscosity (the actual viscosity difference is less than 0.1% [39]). However, the charged pore surfaces lead to streaming potentials and induced electroosmosis that depends on the solution ionic strength. During the manufacturing of track-etched membranes, chemical etching creates acidic functional groups that deprotonate at neutral or basic pH. As mentioned in the introduction, flow through negatively charged nanopores creates a streaming potential, and this potential gives rise to electroosmosis that opposes the pressure-driven flow component. Thus, the spontaneously arising electroosmosis decreases the overall flow velocity in a phenomenon often referred to as electroviscosity [40,41]. However, in aqueous 10 mM KCl, the Debye length is only 3 nm, so the zeta potential, streaming current, and streaming potential are small. The electroosmosis, which is directly proportional to the product of zeta potential and streaming-potential, is insignificant with 10 mM KCl in 30 nm pores. In contrast with a 0.1 mM KCl solution, the Debye length is around 30 nm, and electroosmotic flow is noticeable and retards pressure-driven flow. This provides strong evidence for negatively charged pores and the spontaneously arising streaming potentials that govern the separations and salt rejections described below.

### 3.2. Single-Salt Studies

**Figure 7:**
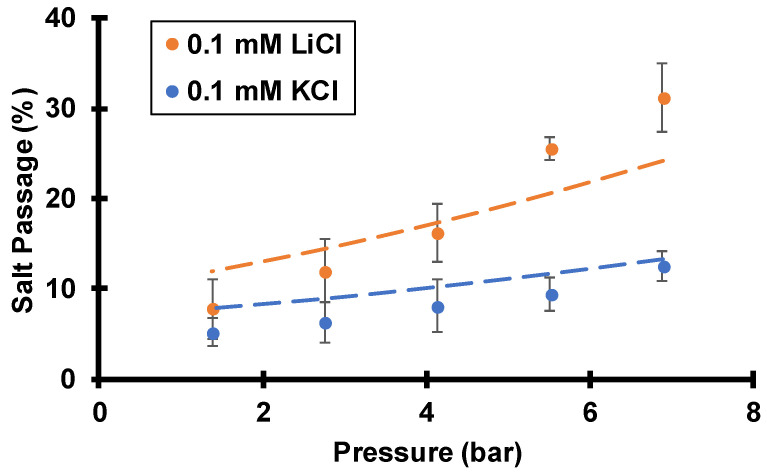
Salt passages during flow of 0.1 mM KCl or 0.1 mM LiCl through track-etched membranes (30 nm pores) using various transmembrane pressures while rotating the membrane at 1000 rpm. Dashed lines are simulated passages assuming a surface charge density of −2.2 mC/m^2^, a pore diameter of 30 nm, and a boundary layer thickness of 19.4 µm, as estimated from the Levich equation.

Figure 7 presents the salt passages during pressure-driven flow of 0.1 mM KCl or 0.1 mM LiCl through track-etched membranes. Filtration of either salt solution shows a passage below 100%, or positive salt rejection (rejection = 100%-passage). The rejections result primarily from the spontaneously arising streaming potentials, not from size exclusion or sieving. The hydrodynamic radii of Li^+^ and K^+^ are around 0.25 and 0.13 nm, respectively, so 30 nm pores will not significantly reject salts via size sieving like nanofiltration or reverse osmosis membranes do.

Because of electrical compensation of the fixed negative charge on pore surfaces, total cation concentrations are around 3 mM within membrane pores (assuming −2.2 mC/m^2^ of surface charge), and only 0.1 mM in the bulk feed solution. This enrichment should enhance passages of cations. However, as Figure 1 shows, the streaming potential gives a cation electromigration flux that opposes the net flux to impede cation passage through the membrane. The cation enrichment and electromigration have opposing effects on cation flux, but under the experimental conditions, electromigration dominates to give positive cation rejection.

Related principles apply to anions. Because of the negative surface charge, anion concentrations are depleted within the membrane compared to the bulk solution (the theoretical Cl^−^ concentration in the nanopores is around 3 µM, so the depletion factor is around 30), and this exclusion should impede anion passage through the membrane. In contrast, the streaming potential induces anion electromigration that is in the same direction as the net flux (Figure 1), and this favors anion transport. The anion depletion and electromigration have opposite effects on transport, and the experimental results show that anion depletion dominates to yield positive anion rejections.

**Figure 8:**
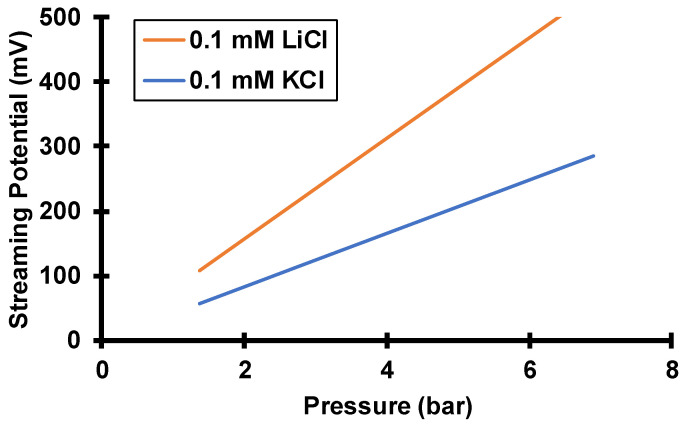
Simulated streaming potentials as a function of transmembrane pressure with either 0.1 mM KCl or 0.1 mM LiCl feed solutions passing through 30 nm pores at different transmembrane pressures. The simulation assumes a surface charge density of −2.2 mC/m^2^ and a boundary layer thickness of 19.4 µm, as estimated from the Levich equation for a rotation rate of 1000 rpm.

As Figure 7 shows, at a given transmembrane pressure LiCl has a higher single-salt passage than KCl, and numerical simulations yield the same behavior. Interestingly, the more mobile cation, K^+^, experiences a lower passage. The diffusion coefficient of K^+^ is nearly twice that of Li^+^ (Table 1). According to Equation (3), electromigration flux is directly proportional to the product of diffusivity, $D_{i}$, and electric field, $-\frac{d\phi }{dx}$. Because of the low diffusivity of Li^+^, the electric field must be higher for a LiCl solution than for a KCl solution to maintain equal cation and anion fluxes (zero-current condition). The simulated streaming potential with LiCl is about twice the streaming potential calculated with KCl (see Figure 8). At the same transmembrane pressure, the Cl^−^ advection flux is the same with either salt solution, but the Cl^−^ electromigration flux is higher in the LiCl solution due to the higher streaming potential. Thus, the net Cl^−^ flux must be higher with the LiCl solution, and LiCl has a higher passage than KCl through these negatively charged track-etched membranes. Note that the streaming potential achieves zero current mostly by decreasing the cation flux. Despite the increase in Cl^−^ flux with LiCl compared to KCl, the streaming potential still must be higher with LiCl to achieve zero current.

Experimental and simulation results (Figure 7) show that both KCl and LiCl passages increase with transmembrane pressure (within the studied pressure range). This increase stems from CP where the rejected solutes accumulate at the membrane/feed interface, so the interfacial salt concentration is higher than the bulk feed-solution concentration. Moreover, CP may increase exponentially with flow rate so the effect of the salt accumulation becomes more evident at higher pressures. Assuming the membrane has a constant intrinsic passage (intrinsic passage is defined as the permeate concentration divided by the interfacial salt concentration, not the feed concentration), observed passages should increase with pressure because interfacial concentrations increase with pressure due to CP. Additionally, the increased salt concentration at the membrane/feed interface weakens Donnan exclusion, and that should lead to an increase in intrinsic passage (see below). Without CP, simulations suggest that salt passages are essentially constant with pressure within the studied pressure range (see Figure S7 of the supplementary materials).

**Figure 9:**
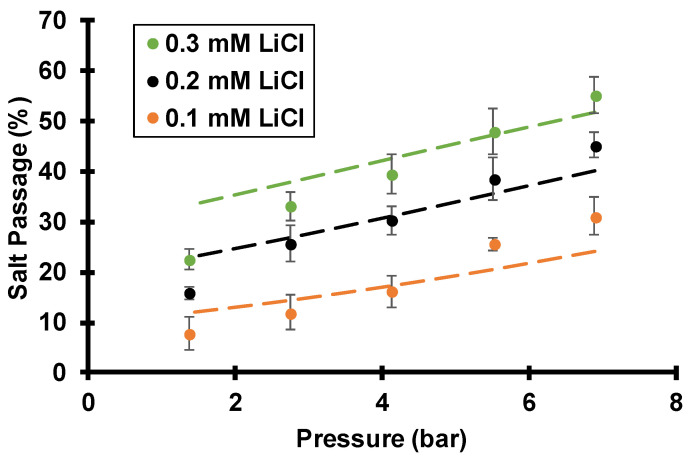
Salt passages during flow of LiCl solutions of various ionic strengths through track-etched membranes (30 nm pores) using various transmembrane pressures and a 1000 rpm rotation rate. Dashed lines are simulated passages assuming a surface charge density of −2.2 mC/m^2^, a pore diameter of 30 nm, and a boundary layer thickness of 19.4 µm, as calculated from the Levich equation.

As Figure 9 shows for LiCl, salt passage increases with the salt concentration in the feed solution. The ratio of the interfacial solution concentration to the bulk concentration, termed the CP factor, should not vary greatly with concentration. Thus, the data in Figure 9 suggest that the membrane’s intrinsic passage increases with the salt concentration in the feed. As mentioned in the introduction, the streaming potential results from an imbalance of cations and anions within the charged pores (Donnan exclusion). As the salt concentration in the feed solution increases, the extent of Donnan exclusion of anions declines so the streaming potential decreases to give higher salt passages. For Li^+^ ions, the increase in bulk salt concentrations in Figure 9 should give minimal increases in the Li^+^ concentration within the nanopores (most of the Li^+^ in the pores is electrically compensating the surface charge). Therefore, the smaller streaming potential is the dominant variable that leads to less electromigration flux (electromigration flux is proportional to the product of concentration and electric field) to enhance the Li^+^ passage. For Cl^−^ ions, less Donnan exclusion should increase the Cl^−^ concentration inside the membrane several fold because Cl^−^ ions are heavily excluded from the negatively charged membrane pores. According to Equation (3), both Cl^−^ advection and electromigration flux should increase at higher ionic strength because they are both proportional to concentration. Despite the smaller electric field, Cl^−^ passage increases with bulk salt concentrations due to less exclusion.

The above single-salt studies elucidate the effects of opposing flow and electric field on salt passage. Although the cation hydrodynamic diameter is two orders of magnitude smaller than the pore diameter (the hydrodynamic radii of Li^+^ and K^+^ are around 0.25 and 0.13 nm, respectively, and the pore diameter is 30 nm), opposing flow and electric field can lead to salt rejections. While the LiCl and KCl salt passages only show minor differences around 10% in Figure 7, mixed-salt studies in which both Li^+^ and K^+^ are present in the feed solution show drastic difference in ion passages (see below).

### 3.3. Mixed-Salt Studies

Section 3.2. shows that flow through 30 nm, negatively charged polycarbonate track-etched membranes leads to single-salt rejection in dilute solutions. As mentioned, these rejections result from the spontaneously arising streaming potentials that give rise to electromigration flux, which is in the opposite direction of advection for cations. In fact, the electromigration velocity retards more than 99% of the advection velocity for cations (see Section S3, Tables S1 and S2 in the supplementary materials) in these single-salt studies.

When considering solutions of mixed salts, one might start by theoretically examining the case in which an infinitely small fraction of LiCl is replaced by KCl (the feed solution ionic strength is kept constant). The electromigration velocity still retards more than 99% of the advection velocity for Li^+^, but the K^+^ electromigration velocity magnitude exceeds its advection velocity and is about twice the Li^+^ electromigration velocity (electromigration velocity is proportional to diffusivity, Equation (3)). In other words, simulation suggests that the K^+^ electromigration velocity exceeds its advection velocity by almost 100% in magnitude. In this case, the Li^+^/K^+^ separation becomes very effective as K^+^ passage will approach 0 at sufficiently high transmembrane pressures (see below). The following sections discuss this phenomenon in more detail and provide experimental and simulation examples.

#### 3.3.1. Effect of Rotation Rate (Concentration Polarization)

**Figure 10:**
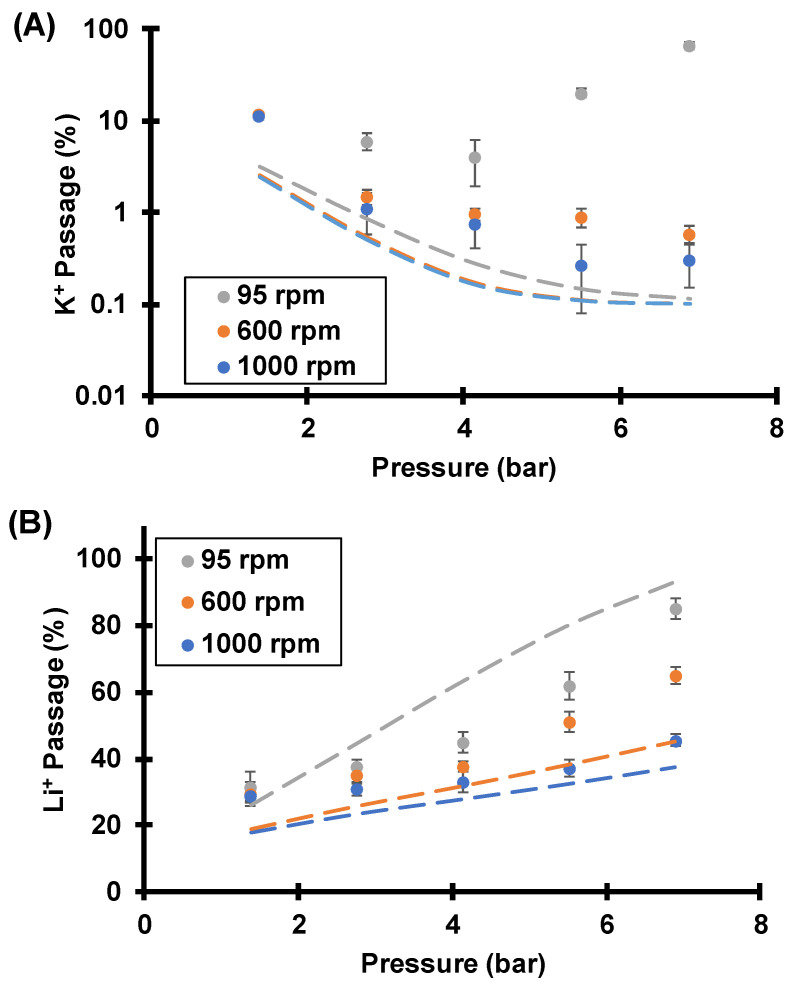
K^+^ (**A**) and Li^+^ (**B**) passages during flow of a 0.05 mM KCl, 0.05 mM LiCl mixture through track-etched membranes (30 nm pores) using various transmembrane pressures and membrane-rotation rates. Note that the upper plot uses a log-scale *y*-axis. Dashed lines are simulated passages assuming a surface charge density of −2.2 mC/m^2^, a pore diameter of 30 nm, and a boundary layer thickness that is calculated from the Levich equation (Table 1). The simulation also incorporates a 0.1% membrane defect area.

**Figure 11:**
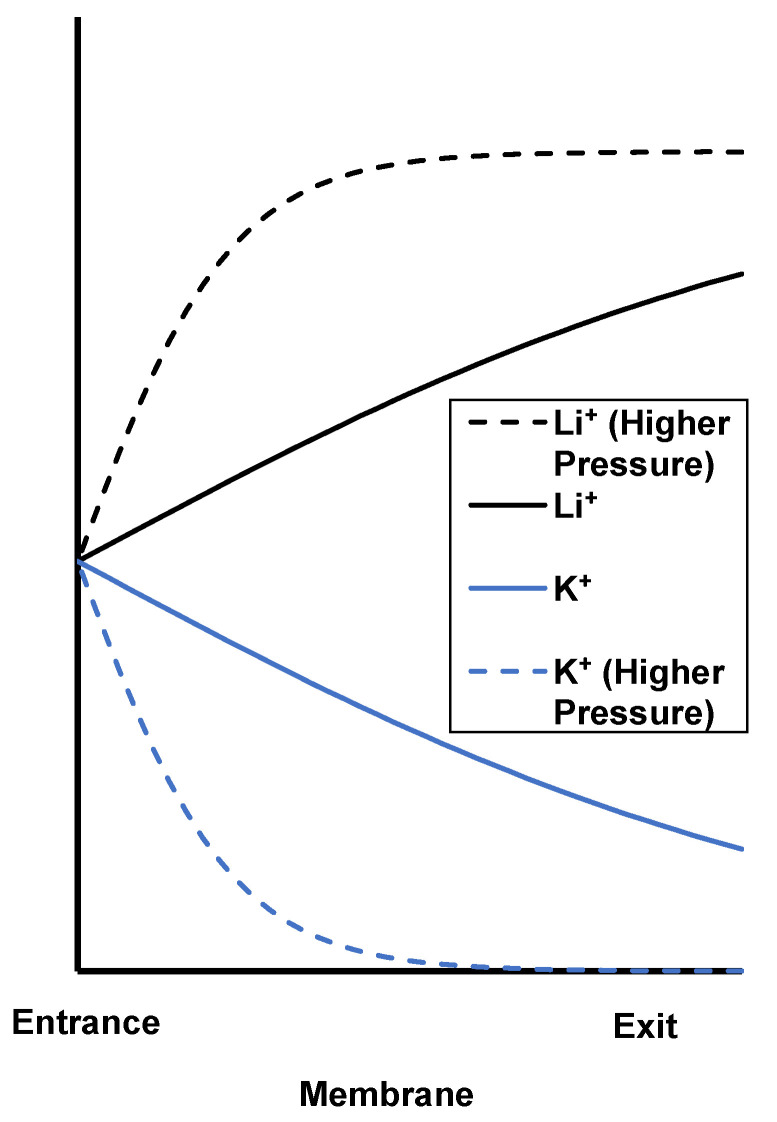
Qualitative K^+^ and Li^+^ concentration profiles within a nanopore. Dashed lines are profiles at a higher transmembrane pressure.

Figure 10 shows Li^+^ and K^+^ passages under various transmembrane pressures and rotation rates during flow of a 0.05 mM LiCl, 0.05 mM KCl mixture through track-etched membranes with 30 nm pores. In most cases, K^+^ passage decreases with transmembrane pressure while Li^+^ passage increases. Thus, the separation is impressive because at high rotation rates both the Li^+^/K^+^ selectivity and Li^+^ passage increase with flow rate, breaking the selectivity–permeability tradeoff, as we saw previously [24]. As Figure 2 shows, the streaming potential retards K^+^ more than Li^+^ because K^+^ has a higher electrophoretic mobility. With sufficiently dilute feed solutions and strong streaming potentials, the K^+^ electromigration velocity exceeds its advective velocity in magnitude. Moreover, the magnitude of the difference between the K^+^ electromigration and advective velocities increases with increasing transmembrane pressure. This leads to a more rapid K^+^ depletion within the membrane (Figure 11) and a smaller K^+^ passage at higher pressures. Thus, the K^+^ passage tends toward 0 as transmembrane pressure, or flow rate, increases. Decreasing the fluid temperature could also facilitate rapid K^+^ depletion. As temperature decreases, the ion diffusion coefficient decreases [42], and the concentration gradient becomes “steeper” at a constant diffusive flux (diffusive flux is proportional to concentration gradient and diffusion coefficient, see Equation (3)). However, chilling the fluid would require refrigeration and add to the energy cost.

As the K^+^ concentration in the membrane pore declines, Li^+^ must compensate the membrane fixed charged to maintain electroneutrality. Thus, increased K^+^ depletion within the nanopores at high flow rates leads to more Li^+^ in the nanopores (Figure 11). Partly for this reason, Li^+^ passage increases while K^+^ passage decreases at higher flow rates.

Unfortunately, the lowest K^+^ passage obtained experimentally was 0.3%, whereas numerical simulations (not shown) predict much smaller values at the highest pressures (K^+^ passage <0.001%). The unwanted experimental K^+^ passage could result from membrane defects and imperfect taping by the “mask”. In Figure 10, the simulation incorporates an additional 0.1% membrane defect area that has 100% ion passage, and the experimental results agree qualitatively well with simulations at 600 and 1000 rpm. For 95 rpm, there is a significant mismatch between experimental data and simulations, which suggests that CP is greater than the simulation suggests. Section S2 in the supplementary materials uses increased boundary layer thicknesses and an optimized surface charge to better fit the trends in the data. For 95 rpm, simulations with the increased boundary layer thickness (see Figure S4) show an increase in the K^+^ passage at the highest pressures and a better fit to the experimental data. As mentioned in the description of the simulations, higher than expected CP might result from imperfect sealing of the tape mask or clustering of pores to give an increased local flow velocity.

Despite the relatively poor agreement between simulations and experiment in Figure 10, numerical simulations still provide insights into these experimental results. As the membrane rotation rate decreases, both K^+^ and Li^+^ passages increase in experimental results and in simulations. Clearly, CP plays an important role in selectivities. For example, at 6.9 bar of transmembrane pressure, the experimental Li^+^/K^+^ selectivity decreases from 150 at 1000 rpm to 1.3 at 95 rpm, a decline of two orders of magnitude. Table S3 of the supplementary materials presents the data in Figure 10 along with selectivities. These results show the importance of using a rotating membrane cell to explore the impact of CP.

The effects of CP on Li^+^/K^+^ selectivities are two-fold. First, the rejected ions that accumulate near the membrane entrance increase the local ionic strength, and the streaming potential decreases because Donnan exclusion weakens. With severe CP at low rotation rates, the streaming potential is not large enough to give a K^+^ electromigration velocity that exceeds its advection velocity in magnitude, and the K^+^ passage is relatively high. Furthermore, in some cases the K^+^/Li^+^ concentration ratio at the membrane surface can significantly increase above the bulk ratio because K^+^ is rejected more by the membrane. For example, in Figure 10 at 6.9 bar of pressure and a 95 rpm rotation rate, the simulated K^+^/Li^+^ concentration ratio at the membrane surface is 1.9, whereas the bulk ratio is 1 (see Figure S8 in the supplementary materials). This inherently decreases selectivity, and it also may lead to lower streaming potentials as the next section shows.

#### 3.3.2. Effect of Feed K^+^/Li^+^ Molar Ratios on Separations

**Figure 12:**
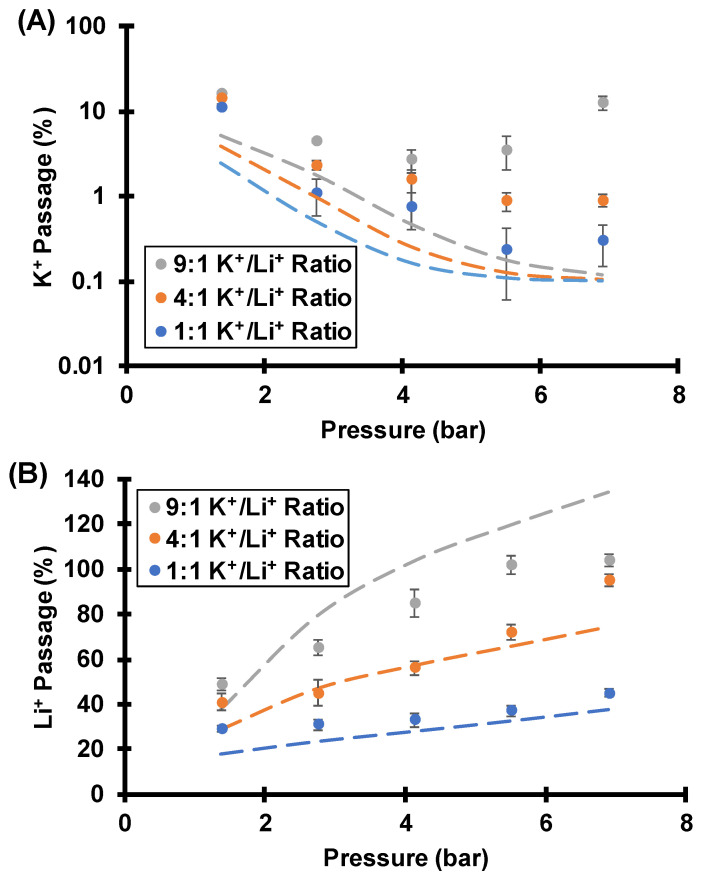
K^+^ (**A**) and Li^+^ (**B**) passages during flow of various 0.1 mM ionic strength KCl and LiCl mixtures through track-etched membranes (30 nm pores) using various transmembrane pressures and a 1000 rpm rotation rate. Dashed lines are simulated passages assuming a surface charge density of −2.2 mC/m^2^, a pore diameter of 30 nm, and a boundary layer thickness of 19.4 µm. The simulation also incorporates a 0.1% membrane defect area. Table S4 in the supplementary materials gives the experimental data and selectivities.

CP can alter the K^+^/Li^+^ concentration ratio at the membrane surface and decrease selectivities. Even without CP, however, the K^+^/Li^+^ ratio in the feed solution affects selectivity. Figure 12 shows that as the K^+^/Li^+^ concentration ratio in the feed solution increases, both K^+^ and Li^+^ passages increase. These results suggest that the streaming potential decreases with a larger K^+^/Li^+^ feed ratio. The ionic strength (0.1 mM) is constant in all the experiments and simulations in Figure 12, so the Donnan exclusion (imbalance of cations and anions within the nanopores) should be the same in all cases. Thus, changes in total cation to anion ratios in the membrane should not occur for the conditions of Figure 12 and should not decrease the streaming potential when the K^+^/Li^+^ feed ratio changes at a constant ionic strength. However, as mentioned in Section 3.2, the magnitude of the streaming potential depends on the cation. The electromigration flux is proportional to the product of diffusivity and electric field, so the electric field needed to give zero current is smaller with K^+^ than Li^+^ because K^+^ has a higher electrophoretic mobility than Li^+^. Thus, as the K^+^/Li^+^ concentration ratio in the feed solution increases, the streaming potential decreases to give higher ion passages. As discussed above, the key to obtaining high K^+^/Li^+^ selectivities is to achieve a streaming potential that gives a K^+^ electromigration velocity that exceeds its advection velocity in magnitude. With a high K^+^/Li^+^ concentration ratio in the feed solution, the streaming potential may not give enough K^+^ electromigration velocity to completely offset advection, and the K^+^ passage is undesirably high.

Unfortunately, with a 9:1 K^+^/Li^+^ ratio in the feed solution and high transmembrane pressures, the experimental K^+^ passage is much larger than the simulated value. Simulations with a larger boundary layer thickness fit the data better (see Figure S5 in the supplementary materials) and suggest the actual extent of CP is again larger than what the Levich equation predicts.

#### 3.3.3. Effect of Feed Ionic Strength

**Figure 13:**
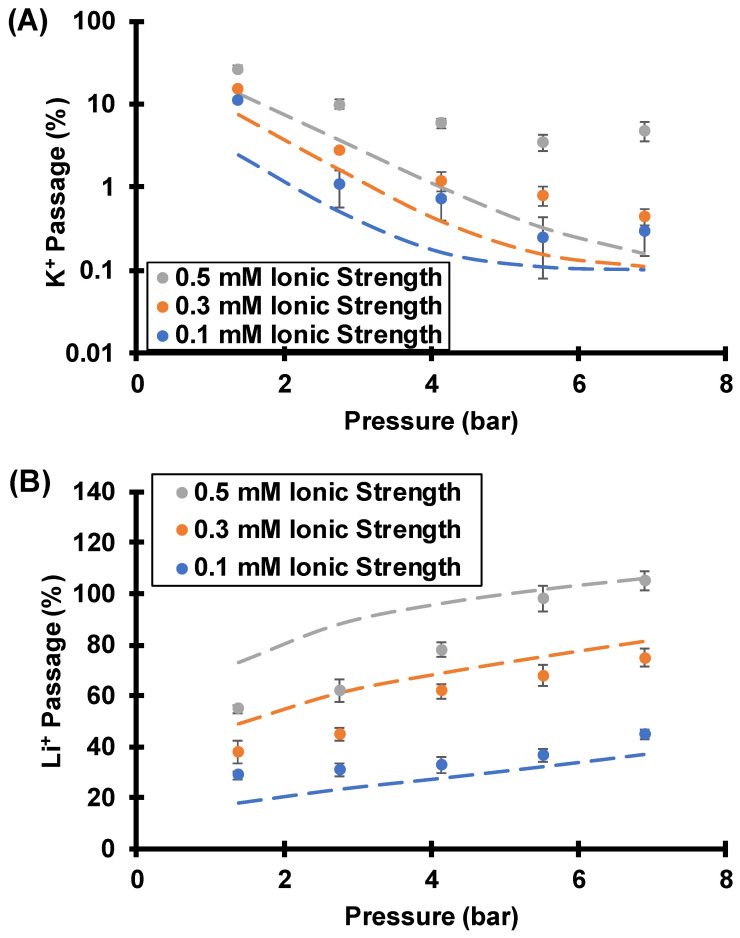
K^+^ (**A**) and Li^+^ (**B**) passages during flow of equimolar KCl and LiCl mixtures at various ionic strengths through track-etched membranes (30 nm pores) using various transmembrane pressures and a 1000 rpm rotation rate. Dashed lines are simulated passages assuming a surface charge density of −2.2 mC/m^2^, a pore diameter of 30 nm, and a boundary layer thickness of 19.4 µm. The simulation also incorporates a 0.1% membrane defect area. Figure S6 shows fits to the data with a higher boundary layer thickness.

In addition to CP and the Li^+^/K^+^ ratio in the feed solution, the ionic strength of the feed solution will affect the Li^+^/K^+^ selectivities by altering exclusion of anions from the membrane pores. In short, ion passages increase with feed ionic strength (Figure 13), and the separations become less effective. For example, at 6.9 bar of transmembrane pressure and a rotation rate of 1000 rpm, the Li^+^/K^+^ selectivity decreases from 150 to 22 as the solution ionic strength increases from 0.1 mM to 0.5 mM. Thus, high selectivities are only possible in dilute solutions. Table S5 in the supplementary materials provides the data in Figure 13 and lists the selectivities.

At sufficiently high ionic strength, the streaming potential becomes negligible because the electrical double layer no longer spans the pore. This work employs pores with a diameter of 30 nm, which limits the feed-solution ionic strength to below 1 mM (10 nm Debye length) to achieve high selectivity. However, practical separations typically occur in much more concentrated solutions such as 1 M salt (0.3 nm Debye length), where a pore diameter of 0.3 nm is required for strong anion exclusion. As mentioned in Section 3.3, effective separations occur at large flow velocities within the nanopores, and it is difficult to obtain such high flow velocities with a pore diameter less than 0.3 nm (for 0.3 nm pores, according to the Hagen–Poiseuille equation, one needs 70,000 bar of transmembrane pressure to get the same maximum flow velocity that was used with 30 nm pores in this work; such high pressures are clearly not feasible). Unfortunately, although the separation mechanism examined in this work can separate among monovalent ions in dilute solutions, it does not apply at higher concentrations. Applied potentials can provide similar separations at higher concentrations, but this requires relatively high currents that lead to a large power consumption [43].

#### 3.3.4. Comparison to Other Studies

As mentioned in the introduction, selective separations among ions that have the same charge are more difficult than monovalent/multivalent ion separations because Donnan exclusion does not separate ions that have the same charge. This work examines the use of opposing flow and electromigration for separating ions that have the same charge. Many other studies have employed different mechanisms/strategies for such separations, and Table 2 summarizes some of the results.

The studies listed in Table 2 as well as others demonstrate that membrane processes show promising selectivities for industry-scale ion separations. The unique feature of this research is that high selectivities do not require modification of membranes with highly selective functional groups, precise control of sub-nanometer pore sizes, or complicated experimental setups that include electrodes. However, Li^+^/K^+^ separations that utilize spontaneously arising streaming potentials require low ionic strengths, relatively high Li^+^/K^+^ molar ratios, and minimal concentration polarization.

## 4. Conclusions

Pressure-driven flow through negatively charged polycarbonate track-etched membranes gives rise to spontaneously arising streaming potentials across the nanopores. These potentials induce ion electromigration that leads to salt rejections, even though the pore size (30 nm diameter) is two orders of magnitude larger than the hydrated diameters of the ions. The streaming potential depends on the solution ionic strength, membrane surface charge density, and more interestingly, the choice of salt. Because Li^+^ has a smaller diffusivity than K^+^, flow of a LiCl solution through the membrane yields a larger streaming potential than flow of a KCl solution. This larger streaming potential increases the net flux through the membrane due to its effect on the anion flux.

Spontaneous streaming potentials can lead to remarkable Li^+^/K^+^ selectivities in mixed-salt studies. Because K^+^ has a larger aqueous electrophoretic mobility than Li^+^, electromigration retards K^+^ more than Li^+^, and membranes selectively reject K^+^ more than Li^+^. More importantly, when the magnitude of K^+^ electromigration exceeds its advection, the K^+^ passage approaches 0 at large transmembrane pressures, and the Li^+^/K^+^ separation becomes very effective and can break the permeability-selectivity tradeoff.

Effective separation requires a sufficiently strong streaming potential, and that imposes limitations on the experimental conditions. The ionic strength of the feed solution needs to be low enough to give extensive Donnan exclusion of the anion. The molar ratio of Li^+^ to K^+^ in the feed must be sufficiently large, as large amounts of K^+^ decrease the streaming potential. Finally, CP must be minimized to avoid its deleterious effects on Donnan exclusion and the Li^+^/K^+^ ratio at the membrane surface. Failure to fulfill any of these conditions can significantly weaken the streaming potential, and when the magnitude of K^+^ electromigration no longer exceeds its advection, K^+^ passage is undesirably high.

In natural aqueous resources, the K^+^/Li^+^ wt% ratio is typically larger than 50, therefore the Li^+^/K^+^ selectivity should be larger than 10,000 to obtain battery-grade Li^+^ product (>99.5% wt%). A two-stage separation with a selectivity larger than 100 in each stage should suffice. Unfortunately, when separating ions using flow and streaming potentials, such high selectivity is difficult to achieve with a high K^+^/Li^+^ wt% ratio in the feed or a high ionic strength. However, opposing flow with an externally applied electric field should address these challenges [43] because the electrical potential drop is not limited by ionic strength, feed composition, and CP. However, the applied potential introduces a significant electrical energy cost that may limit commercialization.

This work focuses on Li^+^/K^+^ separations to elucidate the separation mechanism using two ions whose electrophoretic mobilities differ greatly. However, due to the low electrophoretic mobility of Li^+^, the separation mechanism should also apply to the separation of Li^+^ from cations other than K^+^, albeit with lower selectivity. For example, the electrophoretic mobility of Li^+^ is about 20% smaller than that of Na^+^. Thus, in separations based on opposing flow and electromigration, Li^+^ will have a higher passage through the membrane than Na^+^ [43]. Future work could examine Li+ separations from cations other than K^+^.

## Figures and Tables

**Table 1:** Simulation parameters.

Properties	Value
Pore diameter	30 nm
Pore length	6 µm
Membrane surface charge density	−2.2 mC/m^2^
Transmembrane pressure	20–100 psi (1.4–6.9 bar)
Flow velocity within the boundary layer	Obtained from permeability measurements
Fluid dynamic viscosity	0.00089 Pa/s at 25 °C
Flow velocity within the pores	Calculated from Hagen–Poiseuille model
K^+^ diffusion coefficient ^1^	1.96 × 10^−9^ m^2^/s
Li^+^ diffusion coefficient ^1^	1.03 × 10^−9^ m^2^/s
Cl^−^ diffusion coefficient ^1^	2.03 × 10^−9^ m^2^/s
Boundary layer thickness (from Levich equation for K^+^)	19.4 µm @ 1000 rpm 25.1 µm @ 600 rpm 63.1 µm @ 95 rpm

^1^ Literature values at infinite dilution at 25 °C [27].

**Table 2:** Some literature studies that examine membrane-based separations among ions that have the same charge.

Mechanism	Selectivity
Differences in dehydration energy and mobility between ion species	K^+^/Na^+^ ~850 in electrodialysis [44] Sr^2+^/Mg^2+^ ~900 in electric field-assisted nanofiltration [45] K^+^/Li^+^ and Na^+^/Li^+^ selectivities ~ 100 [16]
Molecular recognition	Li^+^/Na^+^ ~280 and Li^+^/K^+^ ~360 in dialysis [46] K^+^/Na^+^ ~20 in conductivity measurements [23]
Opposing flow and electric field	Li^+^/K^+^ ~150 in dead-end filtration (this work) K^+^/Li^+^ ~30 in hybrid electro-baromembrane process [47]

## Data Availability

Suggested Data Availability Statement: The data presented in this study are available on request from the corresponding author. Much of the data is available in the supplementary materials.

## Supplementary Materials

These materials are available online at https://www.mdpi.com/article/10.3390/membranes12060631/s1. Figure S1: SEM images of polycarbonate track-etched membranes; Figure S2: Salt passages during flow of 0.1 mM KCl or 0.1 mM LiCl through track-etched membranes, along with simulation results using a thick boundary layer; Figure S3: Salt passages during flow of LiCl solutions of various ionic strengths through track-etched membranes, along with simulation results using a thick boundary layer; Figure S4: K^+^ and Li^+^ passages during flow of a 0.05 mM KCl, 0.05 mM LiCl mixture through track-etched membranes, along with simulation results using a thick boundary layer; Figure S5: K^+^ and Li^+^ passages during flow of various 0.1 mM ionic strength KCl and LiCl mixtures through track-etched membranes, along with simulation results using a thick boundary layer; Figure S6: K^+^ and Li^+^ passages during flow of equimolar KCl and LiCl mixtures at various ionic strengths through track-etched membranes, along with simulation results using a thick boundary layer; Figure S7: Simulated LiCl or KCl passages through 30 nm pores in single-salt filtration with and without consideration of CP; Figure S8: Simulated Li^+^ and K^+^ concentrations within a boundary layer; Figure S9: A side-view SEM image of a polycarbonate track-etched membrane; Figure S10: Photograph of the experimental setup for filtration through a rotating membrane; Table S1: Simulated streaming potentials and K^+^ electromigration velocities during filtration of a 0.1 mM KCl solution; Table S2: Simulated streaming potentials and Li^+^ electromigration velocities during filtration of a 0.1 mM LiCl solution; Table S3: Experimental K^+^ and Li^+^ passages and Li^+^/K^+^ selectivities during flow of a 0.05 mM KCl, 0.05 mM LiCl mixture through track-etched membranes; Table S4: Experimental K^+^ and Li^+^ passages and Li^+^/K^+^ selectivities during flow of various 0.1 mM ionic strength KCl and LiCl mixtures through track-etched membranes; Table S5: Experimental K^+^ and Li^+^ passages and Li^+^/K^+^ selectivities during flow of equimolar KCl and LiCl mixtures at various ionic strengths through track-etched membranes; References [24,33,48] are cited in Supplementary Materials.
