# Supplementary material for: Ion Separations Based on Spontaneously Arising Streaming Potentials in Rotating Isoporous Membranes
Source: Membranes (Basel). 2022 Jun 18;12(6):631. doi: 10.3390/membranes12060631 (PMC9227078; doi:10.3390/membranes12060631)
Supplement: Supplementary file 1 [file membranes-12-00631-s001.zip › membranes-1754980-supplementary.pdf]

Electronic Supplementary Information for

Ion Separations Based on Spontaneously Arising Streaming Potentials in Rotating Isoporous Membranes

Chao Tang[a], Andriy Yaroshchuk[b,c,], and Merlin L. Bruening[a,d,*]

[a] Department of Chemical and Biomolecular Engineering, University of Notre Dame, Notre Dame, Indiana 46556, United States

[b] ICREA, pg·L.Companys 23, 08010 Barcelona, Spain

[c] Department of Chemical Engineering, Polytechnic University of Catalonia, av. Diagonal 647,08028 Barcelona, Spain

[d] Department of Chemistry and Biochemistry, University of Notre Dame, Notre Dame, Indiana 46556, United States

* Corresponding author: Merlin L. Bruening E-mail: mbruenin@nd.edu

S1. Deviation between the Levich Model and This Work

Levich derived the mass-transfer boundary-layer thickness for a rotating-disk electrode. However, in the original problem, the electrode is impermeable to flow whereas in this work the polycarbonate track-etched membrane is water-permeable. Thus, it is important to justify the use of Levich's equation.

In the Levich problem, the approximate flow velocity normal to the electrode surface v_y was solved analytically [1] and depends on the distance, y, from the electrode:

$$v_y = -0.51\omega^{1.5}(\frac{\mu}{\rho})^{-0.5}y^2 \quad \text{(Eq. S1)}$$

In the above equation, ω is the electrode rotation rate in rad/s, μ is the solution dynamic viscosity, and ρ is the solution density. v_y is negative because the flow velocity is towards the electrode surface. Note that Eq. S1 is an approximation that is only valid for distances within the mass-transfer boundary layer of thickness δ, which is defined by the Levich equation below and depends on the solute diffusion coefficient D.

$$\delta = 1.61D^{1/3}\omega^{-0.5}(\frac{\mu}{\rho})^{1/6} \quad \text{(Eq.S2)}$$

For KCl solution ($D = 2 \times 10^{-9}$ m^2/s), the boundary layer thickness is 63.1 µm at a 95 rpm rotation rate and 19.4 µm at 1000 rpm.

The membrane disk employed in this study is water-permeable, so Eq. S1 no longer applies because at the membrane surface ($y = 0$), there must be some finite value of v_y as long as there is a transmembrane pressure. We define this velocity at the membrane surface as v_o. Kelson and Desseaux derived Eq. S3, which describes the flow velocity profile near the membrane surface considering a permeable rotating disk [2].

$$v_y = v_o - 0.51\omega^{1.5}(\frac{\mu}{\rho})^{-0.5}y^2 \quad \text{(Eq. S3)}$$

Note that v_o is negative for flow moving from the feed to the membrane. With this flow-velocity profile, we can solve for the concentration profile near the membrane surface using the mass-transfer continuity equation (steady state).

$$v_r\frac{\partial c}{\partial r} + \frac{v_\phi}{r}\frac{\partial c}{\partial \phi} + v_y\frac{\partial c}{\partial y} = D\left[\frac{\partial^2 c}{\partial y^2} + \frac{\partial^2 c}{\partial r^2} + \frac{1}{r}\frac{\partial c}{\partial r} + \frac{1}{r^2}\frac{\partial^2 c}{\partial \phi^2}\right] \quad \text{(Eq. S4)}$$

In this equation, v_r is the flow velocity in the radial direction, and v_ϕ is the angular-flow velocity. Due to symmetry, c should not be a function of ϕ. We also assume that the concentration is not a function of r for a large disc. With these simplifications, we obtain

$$v_y\frac{dc}{dy} = D\frac{d^2c}{dy^2} \quad \text{(Eq.S5)}$$

The boundary conditions are:

$$c(y \to \infty)=c_{bulk} \quad \text{(Eq. S6)}$$

$$-D\frac{dc}{dy}\bigg|_{y=0} + c(y=0)v_y(y=0) = 0 \quad \text{(Eq.S7)}$$

For simplicity, Eq. S7 assumes that the membrane rejects 100% of the solute, and that the solute advective flux at the membrane surface is completely compensated by back diffusion. Although more complicated, one could assume rejections less than 100%. We introduce a change of variable and transform Eq. S5 to S8.

$$v_y u = D\frac{du}{dy} \qquad u = \frac{dc}{dy} \tag{Eq.S8}$$

Separating the variables and integrating gives

$$\int_0^y \frac{\left(v_o - 0.51\omega^{1.5}\left(\frac{\mu}{\rho}\right)^{-0.5} y'^2\right)}{D} dy' = \int_{u_0}^{u} \frac{du'}{u'} \tag{Eq. S9}$$

$$exp\left(\frac{v_o}{D}y - \frac{0.51\omega^{1.5}\left(\frac{\mu}{\rho}\right)^{-0.5} y^3}{3D}\right) = \frac{u}{u_0} \tag{Eq. S10}$$

or

$$\left.\frac{dc}{dy}\right|_{y=0} exp\left(\frac{v_o}{D}y - \frac{0.51\omega^{1.5}\left(\frac{\mu}{\rho}\right)^{-0.5} y^3}{3D}\right) = \frac{dc}{dy} \tag{Eq. S11}$$

Employing Eq. S7 leads to

$$\frac{c(y=0)v_y(y=0)}{D} exp\left(\frac{v_o}{D}y - \frac{0.51\omega^{1.5}\left(\frac{\mu}{\rho}\right)^{-0.5} y^3}{3D}\right) = \frac{dc}{dy} \tag{Eq. S12}$$

Note that $v_y(y=0) = v_o$

$$\frac{c(y=0)v_o}{D} exp\left(\frac{v_o}{D}y - \frac{0.51\omega^{1.5}\left(\frac{\mu}{\rho}\right)^{-0.5} y^3}{3D}\right) = \frac{dc}{dy} \tag{Eq. S13}$$

Integrating and employing Eq. S6 gives

$$\frac{c(y=0)v_o}{D}\int_0^\infty exp\left(\frac{v_o}{D}y - \frac{0.51\omega^{1.5}\left(\frac{\mu}{\rho}\right)^{-0.5} y^3}{3D}\right) dy = \int_{c(y=0)}^{c_{bulk}} dc = c_{bulk} - c(y=0) \tag{Eq. S14}$$

or

$$\frac{c_{bulk}}{c(y=0)} = \frac{v_o}{D}\int_0^\infty exp\left(\frac{v_o}{D}y - \frac{0.51\omega^{1.5}\left(\frac{\mu}{\rho}\right)^{-0.5} y^3}{3D}\right) dy + 1 \tag{Eq. S15}$$

The right-hand side of Eq. S15 can be integrated numerically as long as v_o is specified. Assuming v_o of $-40\ \mu m/s$ (negative means the flow is into the membrane), a rotation rate of 1000 rpm (104.7 rad/s), D of 2 x 10^{-9} m^2/s, and $\frac{\mu}{\rho}$ of 0.89 x 10^{-6} m^2/s, $\frac{c_{bulk}}{c(y=0)}$ is around 0.68 or $\frac{c(y=0)}{c_{bulk}} = 1.47$.

If we employ a thin film model and assume a salt rejection of 100%,

$$\frac{c(y=0)}{c_{bulk}} = exp\left(\frac{\delta v_o}{D}\right) \quad \text{(Eq. S16)}$$

and this leads to a boundary layer thickness δ = 19.0 µm. Remarkably, Levich's equation (Eq. S2) gives a boundary layer thickness of 19.4 µm with a rotation rate of 1000 rpm (104.7 rad/s), D of 2 x 10^{-9} m^2/s, and $\frac{\mu}{\rho}$ of 0.89 x 10^{-6} m^2/s. If we change the rotation rate to 95 rpm, Levich's equation gives a boundary layer thickness of 63.1 µm, whereas our calculation using Eq. S15 and Eq. S16 gives 58.0 µm with a flow velocity through the membrane of 40 µm/s . Thus, it is reasonable to employ Levich's equation in our work, even though the membrane is water permeable.

S2. Simulations with More Evident Concentration Polarization

Derivations in section S1 treat the permeable membrane as a homogenous material. However, the pores in polycarbonate track-etched membrane are discrete (Figure S1), and the flow velocity near the pore entrance must increase significantly compared to the bulk flow velocity in the boundary layer (the porosity is about 1%) [3]. The converging flow could increase the extent of concentration polarization (CP). Additionally, the pores are not evenly distributed and in some membrane areas the pores are somewhat clustered so CP may be more pronounced in these areas.

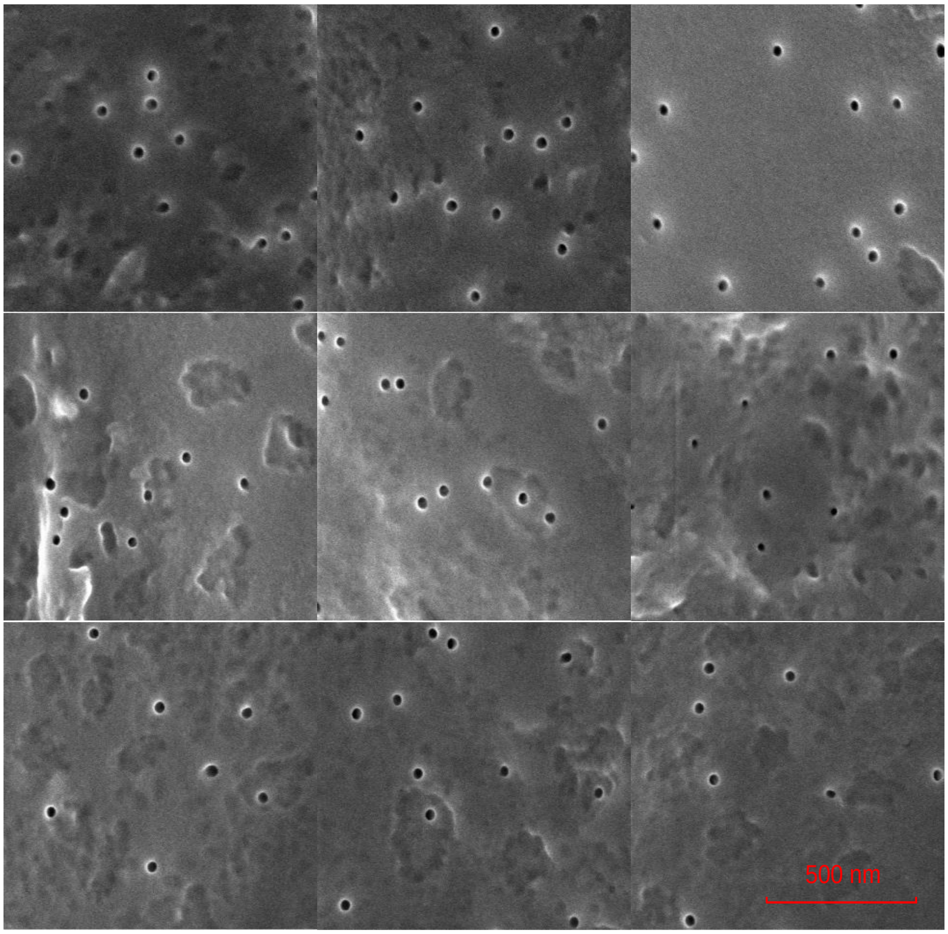

Figure S1. SEM images of polycarbonate track-etched membranes (nominal 30 nm diameter pores). The figure is taken from our previous work [3]. Three images are shown for 3 different membranes, where each row represents images from the same membrane. The scale bar is the same for all images.

Although we do not have direct evidence of elevated CP, the differences between simulations and the data in Figures 7, 9, 10, 12, and 13 suggest that CP may be larger than predicted using the Levich equation. Thus, we also modelled the experimental data in this work with a boundary layer thickness that is 1.9 times that which the Levich equation calculates. We chose the value of 1.9 based on the fit to the data and also used an optimal value of surface charge density. Figures S2-S6 show the results of this modelling. The most important point is that the thicker boundary layers better predict the trends in K^+ passages with mixed salts at high transmembrane pressures. However, we do not have quantitative theoretical justification for the 1.9-fold increase in the boundary layer thickness. Preliminary simulations suggest that the converging flow will only give about a 10% increase in CP at the surface.

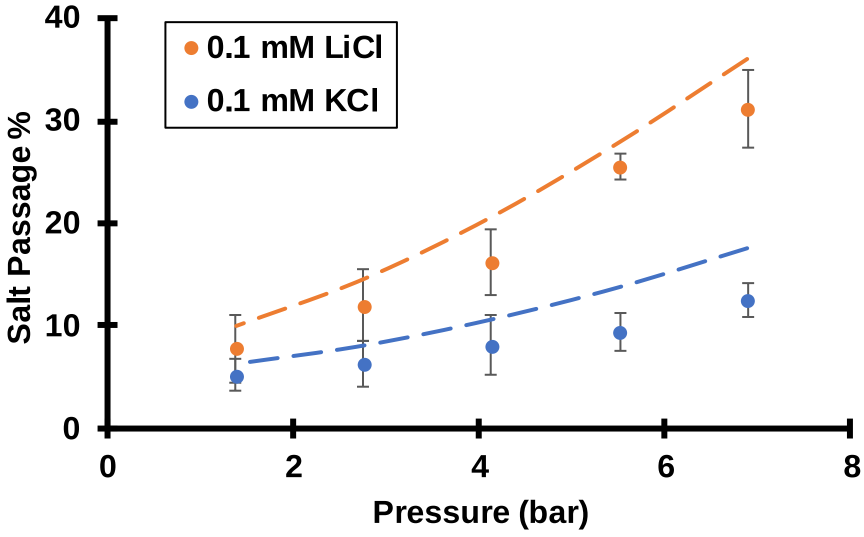

Figure S2. Salt passages during flow of 0.1 mM KCl or 0.1 mM LiCl through track-etched membranes (nominal 30 nm pores) using various transmembrane pressures while rotating the membrane at 1000 rpm. Dashed lines are simulated passages assuming a surface charge density of -2.9 mC/m^2, a pore diameter of 28 nm, and a boundary layer thickness of 38 µm. The boundary layer thickness is 1.9 times that calculated for K^+ from the Levich equation.

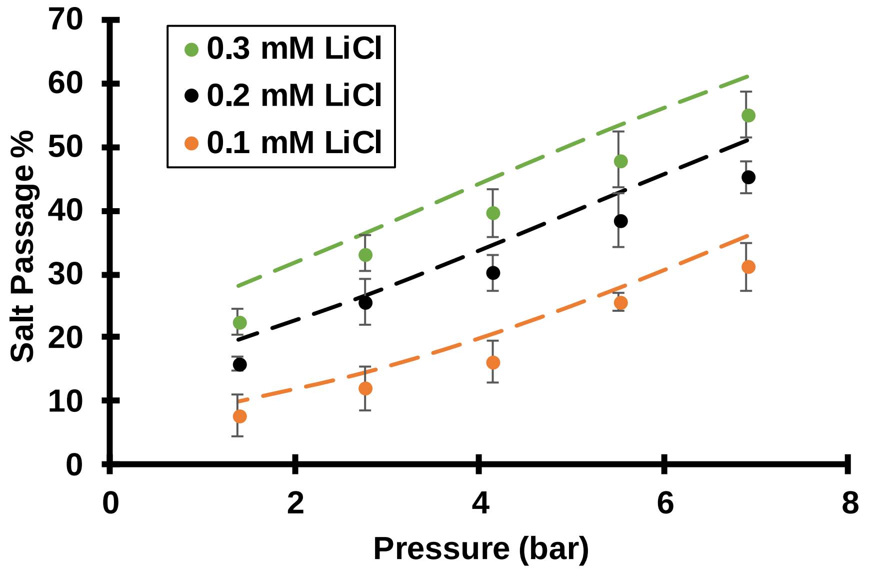

Figure S3. Salt passages during flow of LiCl solutions of various ionic strengths through track-etched membranes (nominal 30 nm pores) using various transmembrane pressures and a 1000 rpm rotation rate. Dashed lines are simulated passages assuming a surface charge density of -2.9 mC/m^2, a pore diameter of 28 nm, and a boundary layer thickness of 38 µm. The boundary layer thickness is 1.9 times that calculated for K^+ from the Levich equation.

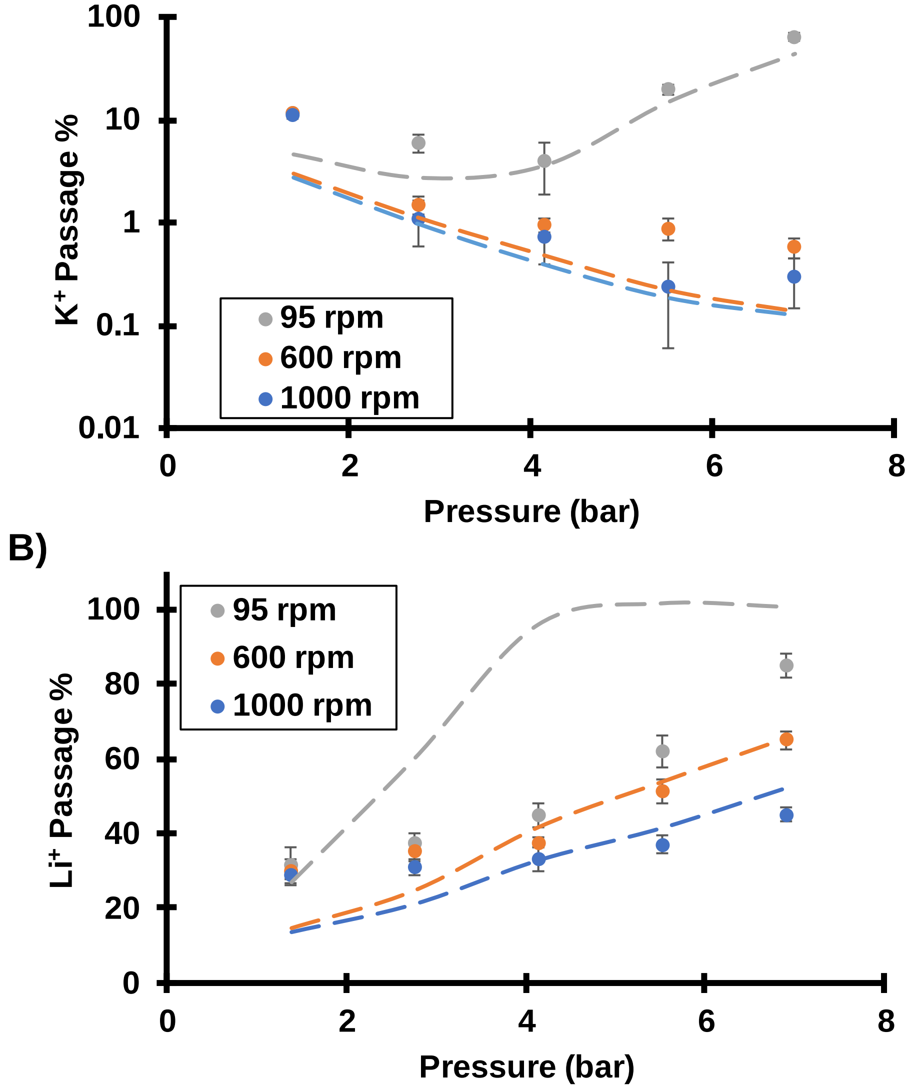

Figure S4. K^+ (A) and Li^+ (B) passages during flow of a 0.05 mM KCl, 0.05 mM LiCl mixture through track-etched membranes (nominal 30 nm pores) using various transmembrane pressures and membrane-rotation rates. Note that the upper plot uses a log-scale y-axis. Dashed lines are simulated passages assuming a surface charge density of -2.9 mC/m^2, a pore diameter of 28 nm, and boundary layer thicknesses that are 1.9 times those calculated for K^+ from the Levich equation. The simulation also incorporates a 0.1% membrane defect area with 100% salt passage.

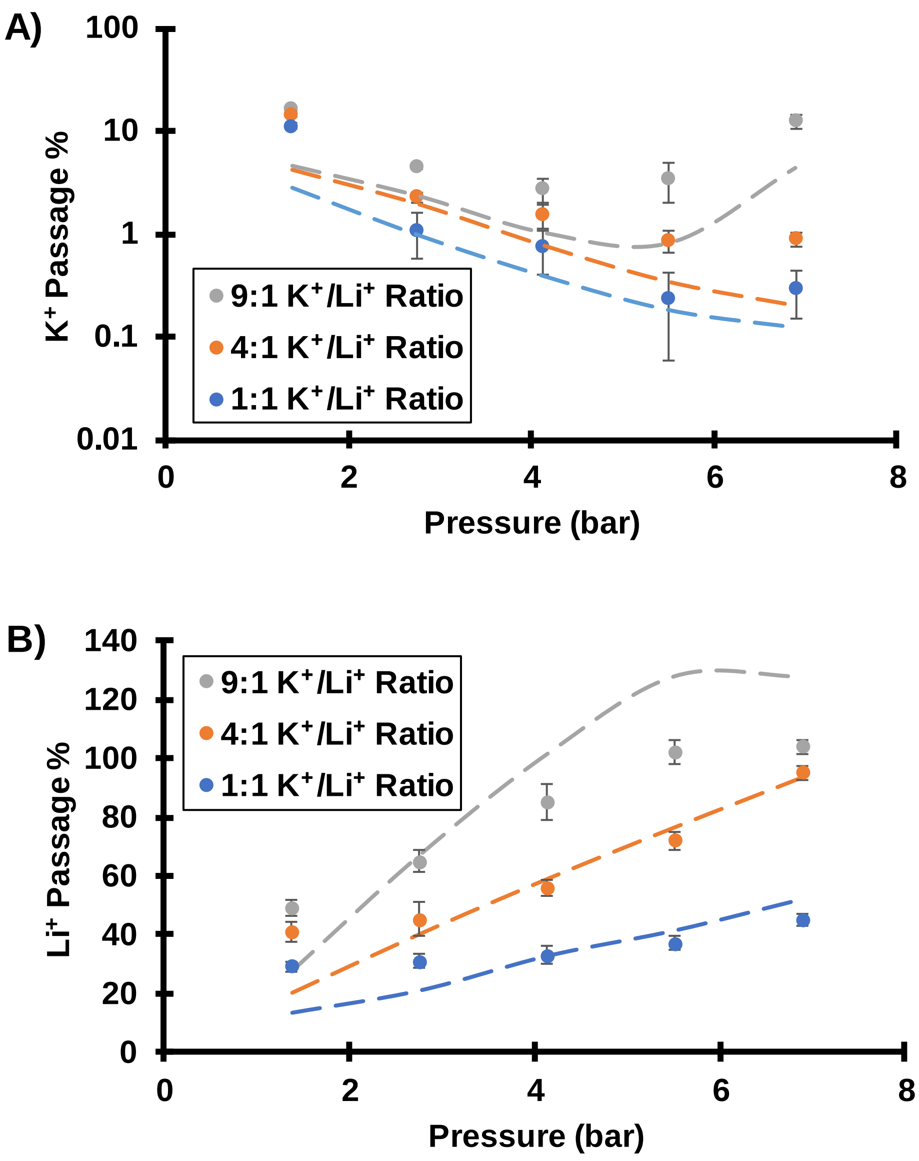

Figure S5. K^+ (A) and Li^+ (B) passages during flow of various 0.1 mM ionic strength KCl and LiCl mixtures through track-etched membranes (nominal 30 nm pores) using various transmembrane pressures and a 1000 rpm rotation rate. Dashed lines are simulated passages assuming a surface charge density of -2.9 mC/m^2, a pore diameter of 28 nm, and a boundary layer thickness of 38 µm. We assume the boundary layer thicknesses are 1.9 times those calculated for K^+ from the Levich equation. The simulation also incorporates a 0.1% membrane defect area with 100% salt passage.

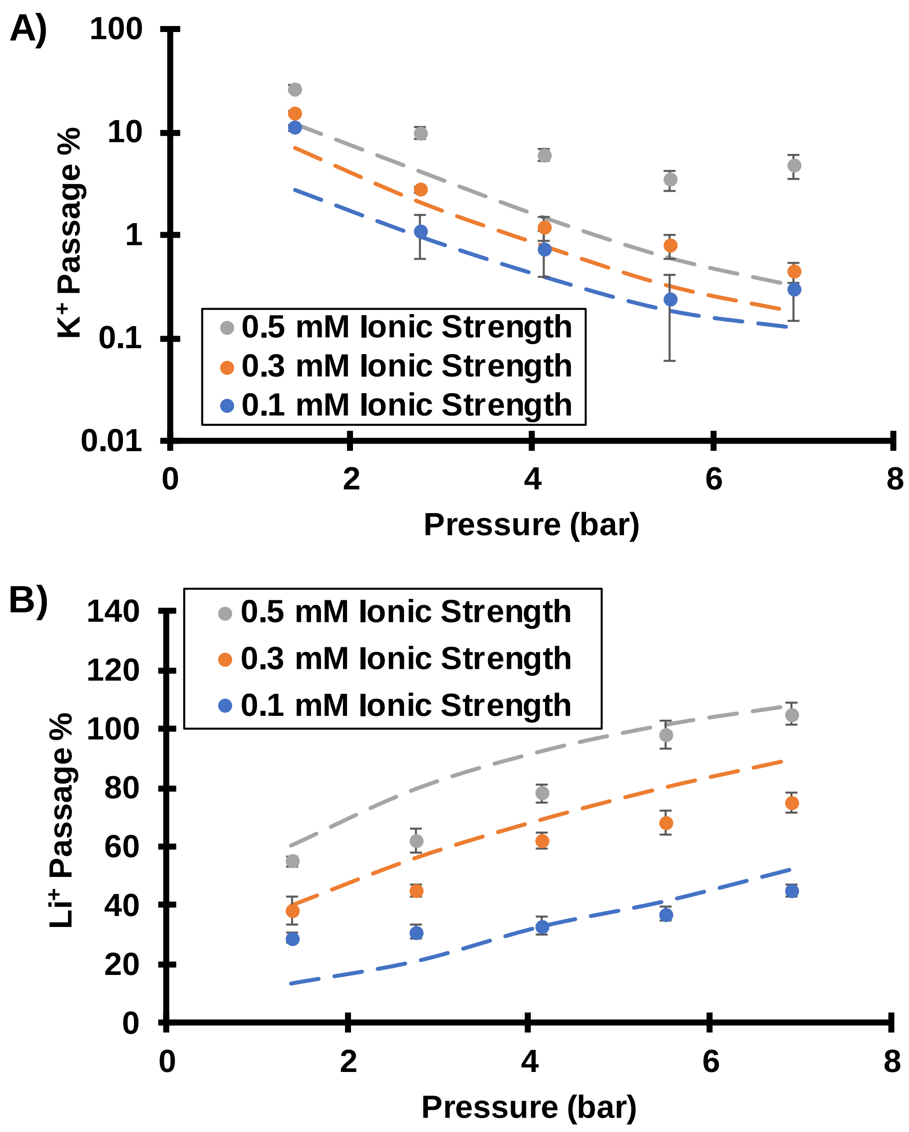

Figure S6. K^+ (A) and Li^+ (B) passages during flow of equimolar KCl and LiCl mixtures at various ionic strengths through track-etched membranes (30 nm pores) using various transmembrane pressures and a 1000 rpm rotation rate. Dashed lines are simulated passages assuming a surface charge density of -2.9 mC/m^2, pore diameter of 28 nm, and a boundary layer thickness of 38 μm. We assume the boundary layer thicknesses are 1.9 times those calculated for K^+ using the Levich equation. The simulation also incorporates a 0.1% membrane defect area.

S3. Streaming Potentials and Electromigration Velocities

Figure 8 in the main text gives the simulated streaming potential with either 0.1 mM KCl or 0.1 mM LiCl feed solutions passing through 30 nm pores at different transmembrane pressures. Interestingly, the streaming potentials give a cation electromigration velocity v_E that retards more than 99% of the advection velocity. Eq. S17 describes the electromigration velocity.

$$v_E = -z_i D_i \frac{F}{RT} \frac{d\phi}{dx} \qquad \text{(Eq. S17)}$$

Here we assume that the electric field is constant in the membrane, so the electric field is simply the streaming potential divided by membrane thickness (6 µm), and Tables S1 and S2 give the calculated electromigration velocities.

Table S1. Simulated streaming potentials and K^+ electromigration velocities as a function of transmembrane pressure with a 0.1 mM KCl feed solution passing through 30 nm pores at different transmembrane pressures. The simulation assumes a surface charge density of -2.2 mC/m^2 and a boundary layer thickness of 19.4 µm.

Pressure (bar)	Flow velocity in nanopores j_v (µm/s)	Streaming potential (V)	Electric field (V/dm)	Electromigration velocity v_E (µm/s)	$\frac{\lvert v_E \rvert}{j_v}$
1.4	726.3	0.0570	-949.6	-724.8	0.998
2.8	1452.5	0.1139	-1898	-1448.6	0.997
4.1	2178.8	0.1707	-2845.7	-2171.9	0.997
5.5	2905.1	0.2275	-3792.3	-2894.4	0.996
6.9	3631.4	0.2842	-4737.8	-3616.0	0.996

Table S2. Simulated streaming potentials and Li^+ electromigration velocities as a function of transmembrane pressure with a 0.1 mM LiCl feed solution passing through 30 nm pores at different transmembrane pressures. The simulation assumes a surface charge density of -2.2 mC/m^2 and a boundary layer thickness of 19.4 µm.

Pressure (bar)	Flow velocity in nanopores j_v (µm/s)	Streaming potential (V)	Electric field (V/dm)	Electromigration velocity v_E (µm/s)	$\frac{\lvert v_E \rvert}{j_v}$
1.4	726.3	0.1082	-1804.1	-723.6	0.996
2.8	1452.5	0.2163	-3604.8	-1445.9	0.995
4.1	2178.8	0.3241	-5401.9	-2166.6	0.994
5.5	2905.1	0.4317	-7194.7	-2885.7	0.993
6.9	3631.4	0.5390	-8982.7	-3602.9	0.992

S4. Simulations of Single-salt Studies without Concentration Polarization (CP)

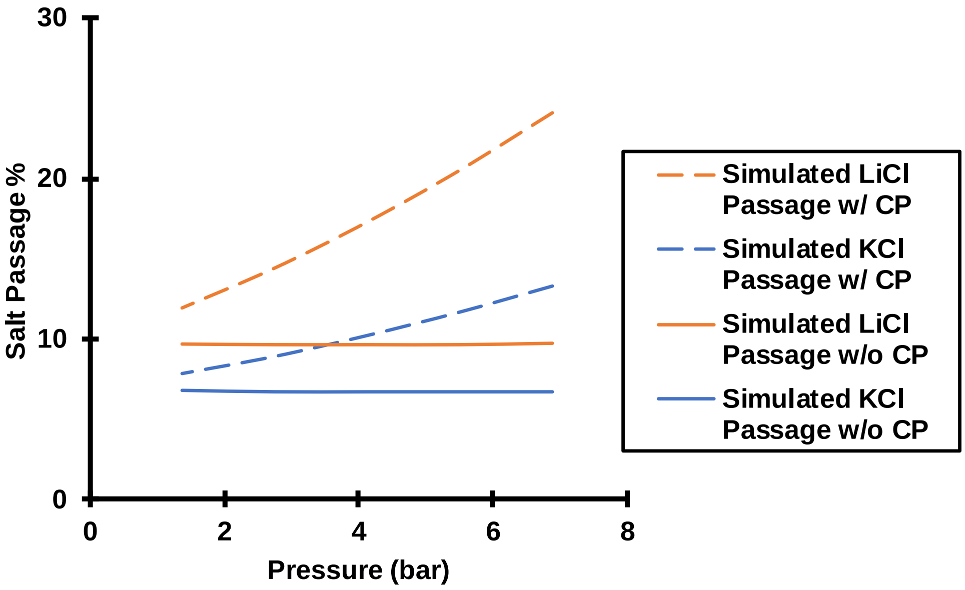

Figure S7. Simulated LiCl or KCl passages through 30 nm pores in single-salt filtration with and without consideration of CP. The solution ionic strength is 0.1 mM, and the surface charge density is assumed to be -2.2 mC/m^2. The boundary layer thickness is 19.4 μm when considering CP.

As Figure S7 shows, simulations suggest that single-salt passages should be approximately constant without any CP. In contrast, the salt passage increases with pressure when CP is considered. As pressure increases, CP becomes more evident and rejected solutes accumulate at the membrane surface. Assuming the membrane intrinsic passage is constant, the observed salt passage should increase with pressure when CP is considered. However, the intrinsic passage should also increase with CP.

S5. Simulated concentration profiles in a boundary layer

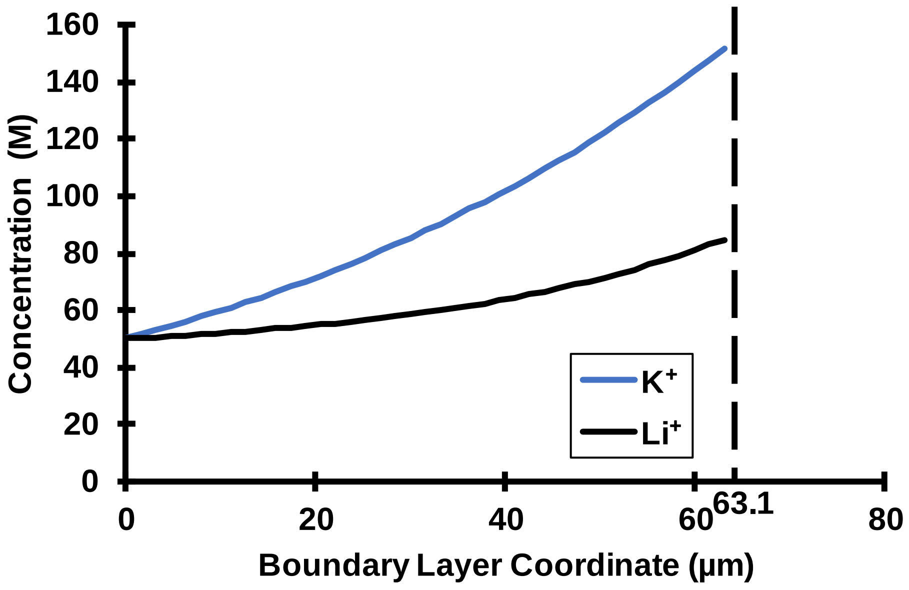

Figure S8. Simulated Li^+ and K^+ concentrations within a 63.1 μm boundary layer during flow a 0.05 mM KCl, 0.05 mM LiCl mixture through track-etched membranes (30 nm pores) at 6.9 bar of transmembrane pressure. The boundary layer thickness is that calculated from the Levich equation for K^+ with rotation at 95 rpm. The simulation assumes a surface charge of -2.2 mC/m^2.

S6. Supporting Experimental Results

Table S3. Experimental K^+ and Li^+ passages and Li^+/K^+ selectivities during flow of a 0.05 mM KCl, 0.05 mM LiCl mixture through track-etched membranes (30 nm pores) at various transmembrane pressures and membrane-rotation rates. These are the experimental results presented in Figure 10 in the main text and in Figure S4.

Pressure (bar)	Rotation (rpm)	K^+ Passage %	Li^+ Passage %	Selectivity
1.4	1000	11.2 ± 0.7	28.9 ± 1.5	2.6 ± 0.2
	600	11.8 ± 0.2	29.6 ± 3.6	2.5 ± 0.3
	95	12.2 ± 0.5	31.8 ± 4.7	2.6 ± 0.4
2.8	1000	1.1 ± 0.52	31.1 ± 2.3	28.3 ± 13.5
	600	1.5 ± 0.28	35.2 ± 2.8	23.5 ± 4.8
	95	6.0 ± 1.2	37.5 ± 2.3	6.3 ± 1.3
4.1	1000	0.75 ± 0.35	33.3 ± 3.2	44.4 ± 21.2
	600	0.95 ± 0.14	37.6 ± 1.5	39.6 ± 6.0
	95	4.6 ± 2.1	45.2 ± 3.2	9.8 ± 4.5
5.5	1000	0.26 ± 0.18	37.0 ± 2.5	142.3 ± 99.0
	600	0.88 ± 0.21	51.4 ± 3.2	58.4 ± 14.4
	95	20.3 ± 2.0	62.4 ± 4.2	3.1 ± 0.4
6.9	1000	0.30 ± 0.15	45.5 ± 1.8	151.7 ± 76.1
	600	0.58 ± 0.12	65.3 ± 2.5	112.6 ± 23.7
	95	65.1 ± 5.7	85.2 ± 3.1	1.3 ± 0.1

Table S4. Experimental K^+ and Li^+ passages and Li^+/K^+ selectivities during flow of various 0.1 mM ionic strength KCl and LiCl mixtures through track-etched membranes (30 nm pores) using various transmembrane pressures and a 1000 rpm rotation rate. These are the results presented in Figure 12 in main text and in Figure S5.

Pressure (bar)	K^+/Li^+ Feed Ratio	K^+ Passage %	Li^+ Passage %	Selectivity
1.4	1:1	11.2 ± 0.7	28.9 ± 1.5	2.6 ± 0.2
	4:1	14.5 ± 0.2	41.2 ± 3.6	2.8 ± 0.3
	9.1	16.5 ± 0.5	49.2 ± 2.8	3.0 ± 0.2
2.8	1:1	1.1 ± 0.52	31.1 ± 2.3	28 ± 14
	4:1	2.3 ± 0.3	45.5 ± 5.8	19.8 ± 3.6
	9.1	4.5 ± 0.3	65.7 ± 3.5	14.6 ± 1.2
4.1	1:1	0.75 ± 0.35	33.3 ± 3.2	44 ± 21
	4:1	1.56 ± 0.45	56.3 ± 4.8	36 ± 11
	9.1	2.7 ± 0.8	85.3 ± 3.5	31.6 ± 9.5
5.5	1:1	0.26 ± 0.18	37.0 ± 2.5	142 ± 99
	4:1	0.88 ± 0.21	72.3 ± 4.2	82 ± 20
	9.1	3.5 ± 1.5	102.5 ± 4.8	29 ± 13
6.9	1:1	0.30 ± 0.15	45.5 ± 1.8	152± 76
	4:1	0.89 ± 0.25	95.0 ± 2.9	107 ± 30
	9.1	12.5 ± 2.1	104.1 ± 2.5	8.3 ± 1.4

Table S5. Experimental K^+ and Li^+ passages and Li^+/K^+ selectivities during flow of equimolar KCl and LiCl mixtures at various ionic strengths through track-etched membranes (30 nm pores) using various transmembrane pressures and a 1000 rpm rotation rate. These are the results presented in Figure 13 in main text and in Figure S6.

Pressure (bar)	Ionic Strength (mM)	K^+ Passage %	Li^+ Passage %	Selectivity
1.4	0.1	11.2 ± 0.7	28.9 ± 1.5	2.6 ± 0.2
	0.3	15.5 ± 0.5	38.6 ± 4.7	2.5 ± 0.3
	0.5	27.2 ± 1.8	55.4 ± 1.6	2.0 ± 0.1
2.8	0.1	1.1 ± 0.5	31.1 ± 2.3	28 ± 14
	0.3	2.8 ± 0.2	45.2 ± 2.3	16.1 ± 1.4
	0.5	9.9 ± 1.3	61.8 ± 4.2	6.2 ± 0.9
4.1	0.1	0.75 ± 0.35	33.3 ± 3.2	44 ± 21
	0.3	1.2 ± 0.38	62.3 ± 2.9	52 ± 17
	0.5	6.3 ± 0.9	78.5 ± 2.5	12.5 ± 1.8
5.5	0.1	0.26 ± 0.18	37.0 ± 2.5	142 ± 99
	0.3	0.82 ± 0.25	68.4 ± 4.2	83 ± 26
	0.5	3.5 ± 0.7	98.3 ± 5.1	28.1 ± 5.8
6.9	0.1	0.30 ± 0.15	45.5 ± 1.8	152 ± 76
	0.3	0.45 ± 0.11	75.2 ± 4.2	167 ± 42
	0.5	4.8 ± 1.4	105.2 ± 3.8	21.9 ± 6.4

S7. Additional Scanning Electron Microscope (SEM) Images

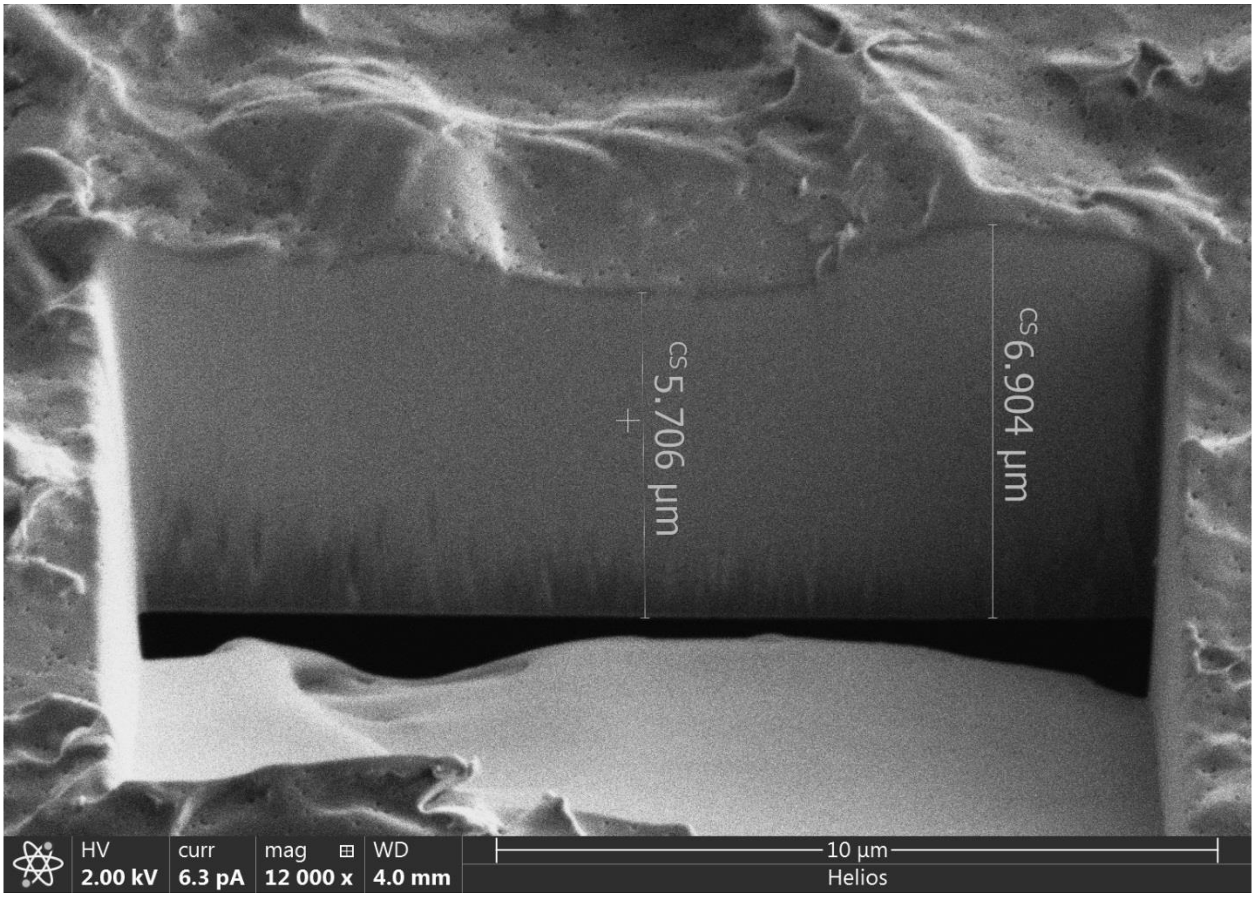

Figure S9. A side-view SEM image of a polycarbonate track-etched membrane (nominal 6 µm thickness). Measurements made in the image took the angle between the focal plane and the membrane surface normal into account. The image was obtained from our previous work [3].

S8. Experimental Setup

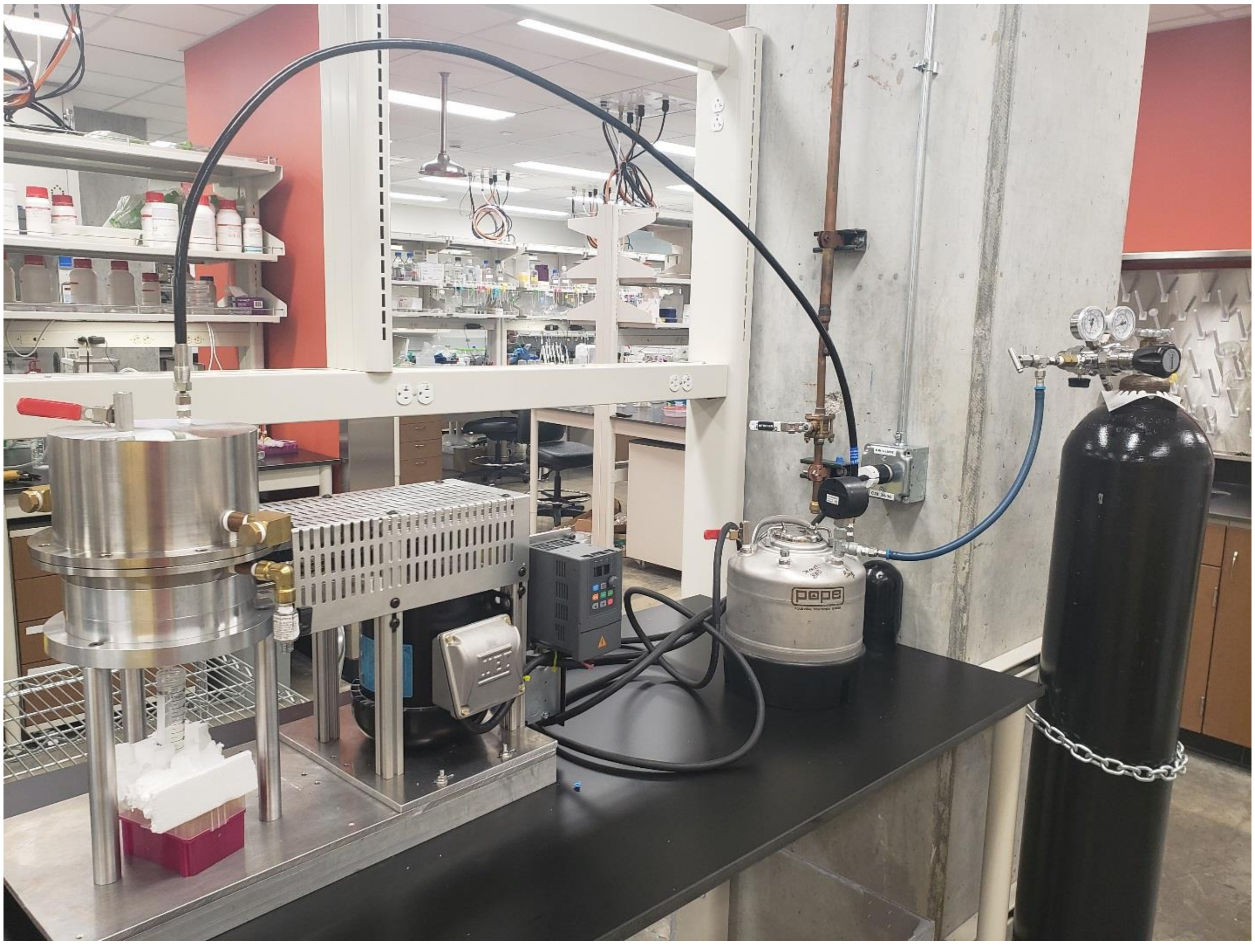

Figure S10. A photograph of the experimental setup for filtration through a rotating membrane.

S9. References

[1] A. J. Bard, L. R. Faulkner, Electrochemical Methods: Fundamentals and Applications, second ed., John Wiley & Sons, Inc., New York, 2001.

[2] N. Kelson, A. Desseaux. Note on porous rotating disk flow. *ANZIAM Journal* **2000**, *42*, C837-C855.

[3] C. Tang, A. Yaroshchuk, M. L. Bruening, Flow through negatively charged, nanoporous membranes separates Li^+ and K^+ due to induced electromigration, *Chem. Commun.* **2020**, *56*, 10954-10957.
